# Phosphorylation and Subcellular Localization of p27Kip1 Regulated by Hydrogen Peroxide Modulation in Cancer Cells

**DOI:** 10.1371/journal.pone.0044502

**Published:** 2012-09-06

**Authors:** Irene L. Ibañez, Candelaria Bracalente, Cintia Notcovich, Ivanna Tropper, Beatriz L. Molinari, Lucía L. Policastro, Hebe Durán

**Affiliations:** 1 Departamento de Micro y Nanotecnología, Comisión Nacional de Energía Atómica, San Martín, Argentina; 2 Consejo Nacional de Investigaciones Científicas y Técnicas, Buenos Aires, Argentina; 3 Escuela de Ciencia y Tecnología, Universidad Nacional de San Martín, San Martín, Argentina; 4 Departamento de Radiobiología, Comisión Nacional de Energía Atómica, San Martín, Argentina; Duke University Medical Center, United States of America

## Abstract

The Cyclin-dependent kinase inhibitor 1B (p27Kip1) is a key protein in the decision between proliferation and cell cycle exit. Quiescent cells show nuclear p27Kip1, but this protein is exported to the cytoplasm in response to proliferating signals. We recently reported that catalase treatment increases the levels of p27Kip1 *in vitro* and *in vivo* in a murine model. In order to characterize and broaden these findings, we evaluated the regulation of p27Kip1 by hydrogen peroxide (H_2_O_2_) in human melanoma cells and melanocytes. We observed a high percentage of p27Kip1 positive nuclei in melanoma cells overexpressing or treated with exogenous catalase, while non-treated controls showed a cytoplasmic localization of p27Kip1. Then we studied the levels of p27Kip1 phosphorylated (p27p) at serine 10 (S10) and at threonine 198 (T198) because phosphorylation at these sites enables nuclear exportation of this protein, leading to accumulation and stabilization of p27pT198 in the cytoplasm. We demonstrated by western blot a decrease in p27pS10 and p27pT198 levels in response to H_2_O_2_ removal in melanoma cells, associated with nuclear p27Kip1. Melanocytes also exhibited nuclear p27Kip1 and lower levels of p27pS10 and p27pT198 than melanoma cells, which showed cytoplasmic p27Kip1. We also showed that the addition of H_2_O_2_ (0.1 µM) to melanoma cells arrested in G1 by serum starvation induces proliferation and increases the levels of p27pS10 and p27pT198 leading to cytoplasmic localization of p27Kip1. Nuclear localization and post-translational modifications of p27Kip1 were also demonstrated by catalase treatment of colorectal carcinoma and neuroblastoma cells, extending our findings to these other human cancer types. In conclusion, we showed in the present work that H_2_O_2_ scavenging prevents nuclear exportation of p27Kip1, allowing cell cycle arrest, suggesting that cancer cells take advantage of their intrinsic pro-oxidant state to favor cytoplasmic localization of p27Kip1.

## Introduction

Cell cycle progression pathways are the endpoint of signaling cascades implicated in cell growth and cell proliferation. Cell cycle is tightly coordinated by sequential assembly and activation of phase-specific protein kinase complexes [1], [2], formed by cyclins and cyclin-dependent kinases (CDKs), which are also regulated by the INK4 proteins and the CDK inhibitors (CDKIs). D-type cyclins are expressed throughout the cycle in response to mitogen stimulation [2]. Cyclin D-CDK4 and cyclin E-CDK2 complexes are required for the passage from G1 to S phase. The CDKI 1B (CDKN1B), also known as p27Kip1, was first identified as a critical negative regulator of CDK2 and G1/S cell cycle progression [2], [3]. The levels of this CDKI are high in quiescent cells, fall in response to mitogenic stimulation, remain at threshold levels in proliferating cells, and increase again when mitogens are withdrawn [2].

In recent years, it was found that p27Kip1 is involved in the regulation of other processes such as cell migration [4] along with cell proliferation, differentiation and apoptosis [5]. Interestingly, this protein can exert both positive and negative functions on these processes [5]. The activities of p27Kip1 are controlled by its concentration, subcellular localization and phosphorylation status [5]. For example, the phosphorylation of p27Kip1 at serine 10 (S10) mediates p27Kip1 exportation to the cytoplasm [6]–[9], the phosphorylation at threonine 198 (T198) stabilizes the protein in the cytoplasm and increases p27Kip1-dependent cell motility [4] and the phosphorylation at threonine 187 (T187) points p27Kip1 as a target for proteolysis by polyubiquitination [9]–[11]. The phosphorylation of other sites of the protein impairs nuclear import of p27Kip1 and enhances the assembly of cyclin D1-CDK4 complex [9], [12]–[15] or initiates the transition of p27Kip1 from inhibitor of cyclin E-CDK2 to substrate for proteolysis [16], [17]. Alterations in p27Kip1 phosphorylation could lead to loss of stability, aberrant function or mislocalization of the protein which, in turn, could contribute to oncogenesis [5], [9]. In this sense, both loss of nuclear p27Kip1 and its cytoplasmic localization have been proposed as prognostic marker for melanoma progression and worse clinical outcome [18].

### Extracellular Environment can Initiate Cell Cycle Division or Arrest by Activating or Deactivating Cyclin-CDK Complexes through Different Pathways

Reactive oxygen species (ROS) are capable of exerting different effects on the cells according to their nature, localization and levels [19]. Particularly, many types of mammalian cells can increase their growth when exposed to moderate levels of hydrogen peroxide (H_2_O_2_) and can induce apoptosis [20], terminal differentiation [21] or cytotoxicity [20] if exposed to high levels of H_2_O_2_. Scavenging of H_2_O_2_ in tumor cells either treated with exogenous catalase or expressing transfected catalase inhibits cell proliferation [22]–[25]. It is well documented that H_2_O_2_ is involved in signal transduction pathways [26], [27], e.g. increased levels of H_2_O_2_ induce mitogenic signals, such as those related to Ras/extracellular signal-regulated kinases 1 and 2 (ERK1/2) pathway, and stress-responsive signals, such as those related to c-Jun N-terminal kinases (JNKs) and p38 mitogen-activated protein kinase (MAPK) pathways [26]–[28]. Moreover, ROS, and in particular H_2_O_2_, were also implied in the modulation of receptor tyrosine kinases (RTK) [29] and phosphatidylinositol 3-kinase (PI3K)/AKT [30] pathways.

It has been reported that fluctuations observed in the intracellular redox state during cell cycle progression could link oxidative metabolic processes to cell cycle regulation [31], [32]. H_2_O_2_ fluctuations along the cell cycle were associated with the regulation of cyclin D1 expression [33]. In contrast, removal of endogenous H_2_O_2_ by overexpression of catalase and glutathione peroxidase induces G0/G1 arrest [25] and decreases cell DNA synthesis [34]. A recent study of our laboratory showed increased levels of p27Kip1 in response to catalase treatment in a murine model of squamous cell carcinoma *in vitro* and *in vivo* [35]. However, the mechanisms involved in this cell cycle protein regulation by H_2_O_2_ have not been fully understood. Considering that p27Kip1 was proposed as a prognostic biomarker for human melanoma [18] and that these tumors exhibited a pro-oxidant behavior due to an imbalance in the antioxidant system [36], [37] and to the melanin deregulation [38], human melanoma cells become an interesting model in order to broaden our previous results on H_2_O_2_ regulation of p27Kip1.

The aim of the present study was to evaluate the effects of the modulation of H_2_O_2_ levels on G1/S transition and, in particular, on the regulation of the CDKI protein, p27Kip1, in human melanoma and melanocyte cell lines. We demonstrated the intracellular relocalization of p27Kip1 after catalase or H_2_O_2_ treatments. This was associated with variations on the levels of phosphorylated p27Kip1 at S10 (p27pS10) and T198 (p27pT198), which play an important role in the regulation of the subcellular localization of this protein. Results on p27Kip1 modulation were extended to other human cancer cell types, colorectal carcinoma and neuroblastoma cells. Our findings can provide a clue to understand the effect of H_2_O_2_ on the modulation of a key regulatory protein of G1/S transition with the consequent effect on cell cycle and cell proliferation.

## Results

### Catalase Treatment Inhibits Melanoma Cell Proliferation by G1 Arrest

**Figure 1:**
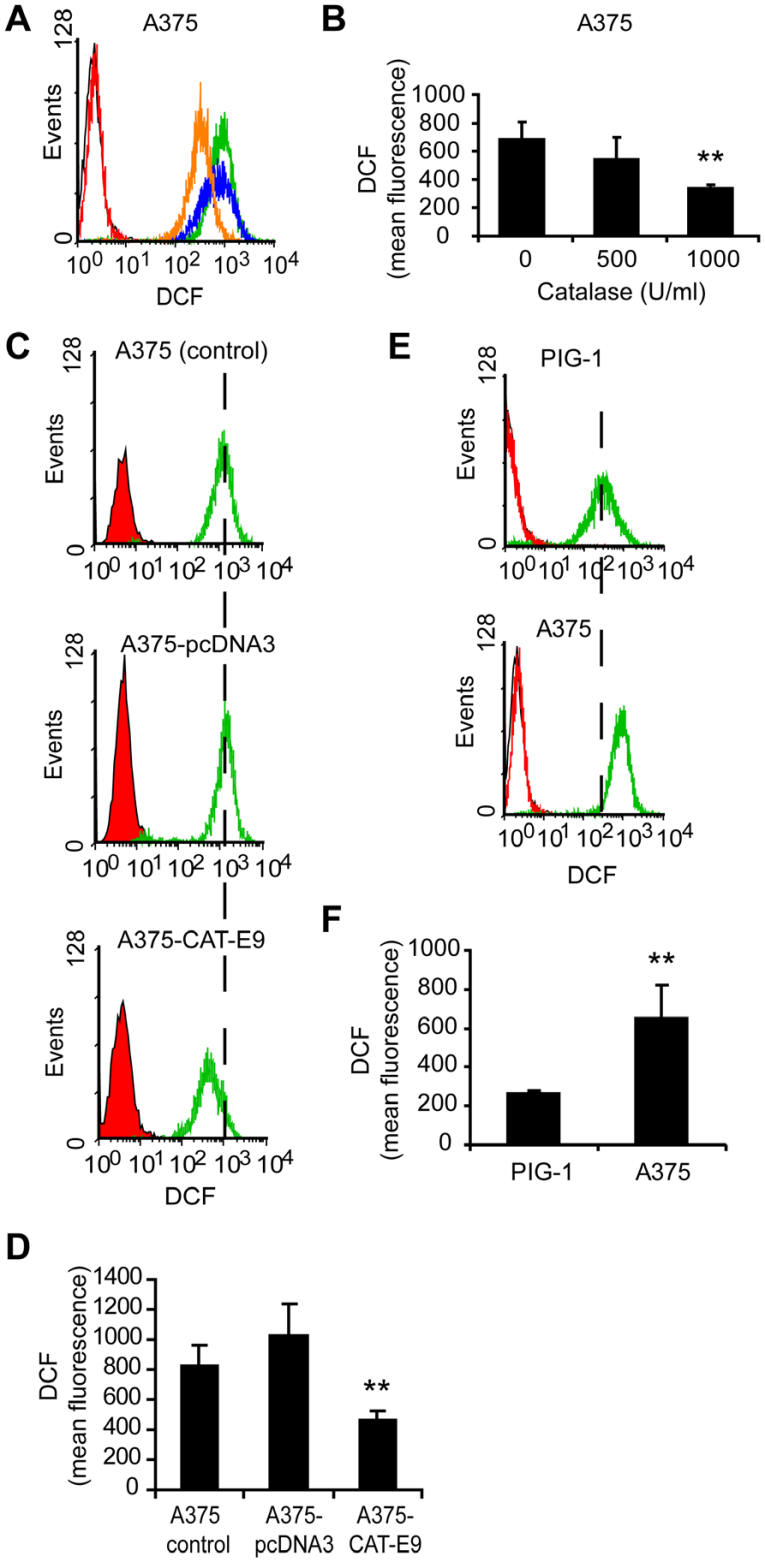
Intracellular ROS levels in melanoma cells and melanocytes determined by DCFH-DA assay. (A–D) Intracellular ROS levels decreased in melanoma cells either when treated with catalase or when overexpressing it. (A) Representative histograms of DCF fluorescence of melanoma cells treated with 500 (blue line) and 1000 (orange line) U/ml catalase or left untreated (green line) for 24 h. Control cells not exposed to DCFH-DA (black line) and control cells treated with catalase just before DCFH-DA incubation (red line). (B) DCF mean fluorescence (arbitrary units) vs. catalase (CAT) dose. Data are expressed as mean ± SD. **p<0.01 vs. untreated cells (0 U/ml catalase). (C) Representative histograms of DCF fluorescence of melanoma cells overexpressing catalase (A375-CAT-E9) and its controls (A375-pcDNA3 and untreated A375 cells). Control cells not exposed to DCFH-DA (black line), control cells treated with catalase just before DCFH-DA incubation (red line) and cells incubated with DCFH-DA (green line). (D) DCF mean fluorescence (arbitrary units) of A375-CAT-E9, A375-pcDNA3 and A375 control cells. Data are expressed as mean ± SD. **p<0.01 vs. A375 control. (E-F) Melanoma cells (A375) exhibited higher levels of intracellular ROS than their non-tumor counterpart (PIG-1 melanocytes). (E) Representative histograms of DCF fluorescence of PIG-1 and A375 cells: control cells not exposed to DCFH-DA (black lines), control cells treated with catalase just before DCFH-DA incubation (red line) and cells incubated with DCFH-DA (green line). (F) DCF mean fluorescence (arbitrary units) of PIG-1 melanocytes and A375 melanoma cells. Data are expressed as mean ± SD. **p<0.01 vs. PIG-1.

**Figure 2:**
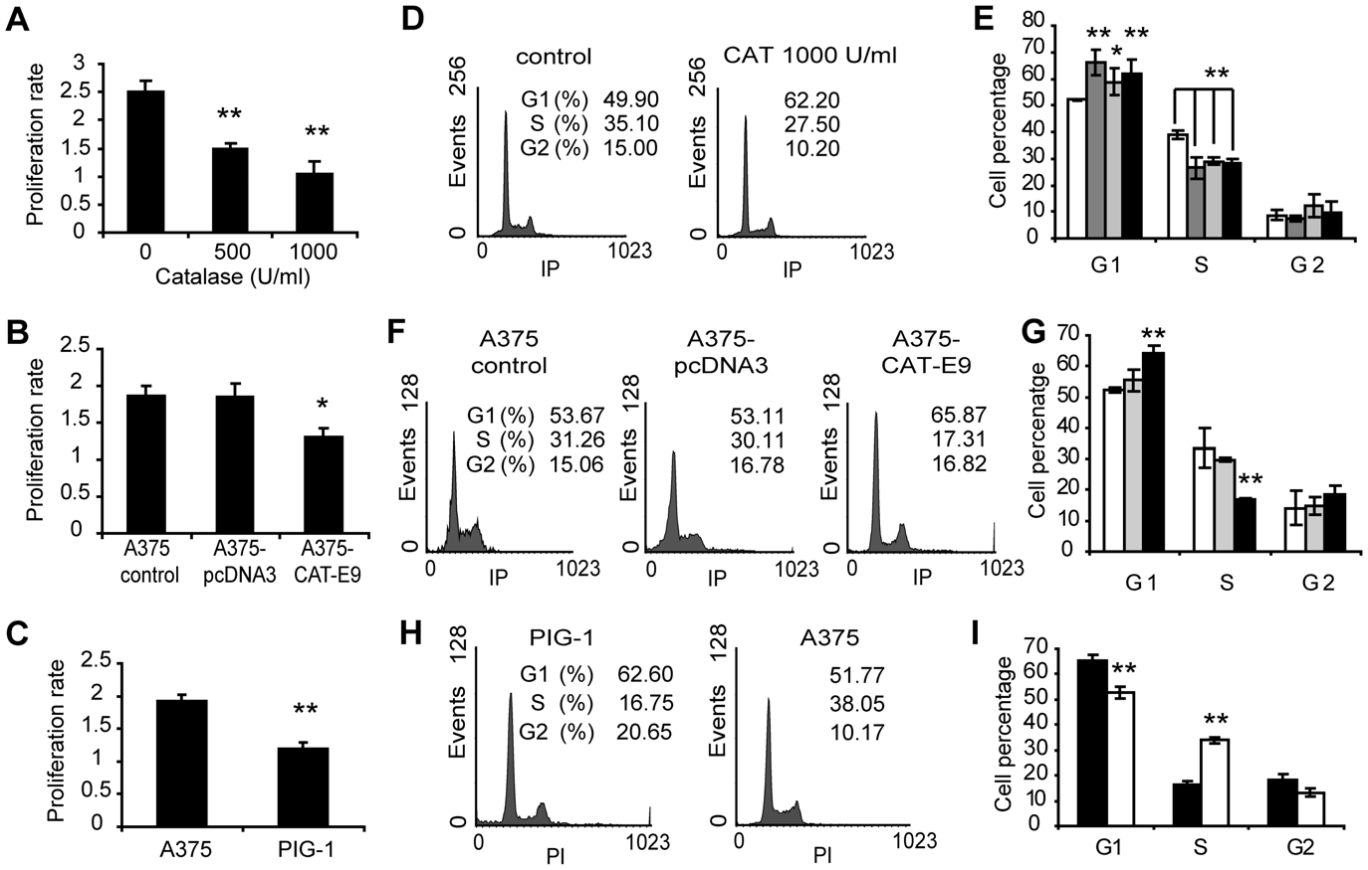
Decreased cell proliferation rate and cell cycle arrest in response to lowered levels of ROS. (A) Cell proliferation rate of melanoma cells treated with catalase for 24 h, relative to control cells, evaluated by the MTT assay. Data are expressed as mean ± SD. **p<0.01 vs. control. (B) Proliferation rate in A375-CAT-E9, A375-pcDNA3 and A375 control cells. Data are expressed as mean ± SD. *p<0.05 vs. A375 control. (C) Proliferation rate of non-tumor (PIG-1) and tumor (A375) cells. Data are expressed as mean ± SD. **p<0.01 vs. A375. (D–I) Cell cycle analysis assessed by flow cytometry after staining with propidium iodide. (D) Representative histograms of DNA content of A375 melanoma cells treated with 1000 U/ml catalase (CAT) during 24 h and A375 control cells. (E) Percentage of melanoma cells in the different phases of the cell cycle in response to CAT treatment. FBS starved cells were used as control of G1 arrest. (□) Untreated control cells, (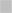) 500 U/ml and (■) 1000 U/ml CAT and (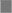) FBS starved cells. Data are expressed as mean ± SD. *p<0.05 and **p<0.01 vs. untreated control. (F) Representative histograms of DNA content of A375-CAT-E9, A375-pcDNA3 and A375 control cells. (G) Percentage of A375-CAT-E9 (■), A375-pcDNA3 (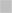) and A375 control cells (□) in the different phases of the cell cycle. Data are expressed as mean ± SD. **p<0.01 vs. A375 control. (H) Representative histograms of DNA content of PIG-1 melanocytes and A375 melanoma cells. (I) Percentage of (■) non-tumor (PIG-1) and (□) tumor (A375) cells in the different phases of the cell cycle. Data are expressed as mean ± SD. **p<0.01 vs. PIG-1 cells.

It has been suggested that cells with a permanent oxidative shift in the redox status may undergo continuous proliferation that could, in turn, be a crucial event in the appearance of the malignant phenotype [23], [39]. In this sense, the production of large amounts of ROS and, in particular, H_2_O_2_, was reported in tumor cell lines [23], [40]. Catalase is an antioxidant enzyme that decomposes H_2_O_2_ in water and oxygen. Taking into account that H_2_O_2_ can diffuse across membranes, the addition of catalase to the culture medium could produce a decrease in the intracellular level of H_2_O_2_ reaching a corresponding lower steady state concentration inside and outside the cell [26], [33], [35]. We validated our model of melanoma cells treated with catalase added to the culture medium by measuring the decrease in the levels of ROS through 2′, 7′-dichlorodihydro-fluorescein diacetate (DCFH-DA) assay (Figures 1A and 1B). This decrease in ROS levels induced by catalase resulted in a significant inhibition (p<0.01) of cell proliferation (Figure 2A). Moreover, A375 cells overexpressing catalase (A375-CAT-E9) displayed low rate of cell proliferation as compared with control cells (Figure 2B). This clone, A375-CAT-E9, showed the lowest intracellular ROS levels of the stable geneticin-resistant clones generated (Figure S1A) and a significant decrease in these levels as compared to cells transfected with an empty plasmid (A375-pcDNA3) or non-transfected (A375 control) (Figures 1C and 1D). These results agree with the higher levels of catalase expression and activity observed in A375-CAT-E9 as compared with control cells (Figure S1B and S1C).

In order to evaluate non-tumor and tumor cells in association with their intracellular ROS levels, we analyzed melanocytes vs. melanoma cells. Figure 2C shows the proliferation rate of PIG-1 melanocytes in comparison to A375 melanoma cells. We confirmed that tumor cells exhibited higher intracellular levels of ROS than their non-tumor counterpart in this melanoma/melanocyte model (Figures 1E and 1F). No significant differences in the levels of ROS and in the proliferation rate (Figure S2) were observed between non treated and heat-inactivated catalase treated cells in the melanoma model. Thus, heat-inactivated catalase was used as control.

A significant G1 cell cycle arrest was found associated with the inhibition of cell proliferation in melanoma cells treated with catalase for 24 h (Figures 2D and 2E). Analogous results were observed for A375-CAT-E9 vs. A375-pcDNA3 or A375 control cells (Figures 2F and 2G). Melanocytes showed higher percentage of cells in G1 phase and a lower percentage in S phase than melanoma cells (Figures 2H and 2I).

Regarding the levels of the cyclins and CDKs involved in G1/S transition, cyclin D1, cyclin E, CDK4 and CDK2, evaluated by western blot, a significant decrease in cyclin D1 levels was observed after H_2_O_2_ removal by catalase treatment or catalase overexpression (Figure S3), in agreement with previous findings [35], [41].

Moreover, the signal for cyclin D1 detected by immunofluorescence was extremely low in the nucleus of cells treated with catalase and a significant decrease of the percentage of positive nuclei was found in these cells as compared with control cells (Figure S4). Melanoma cells exhibited higher amount of both cyclin D1 levels and the percentage of positive nuclei for cyclin D1, assessed by western blot and immunofluorescence than their non-tumor counterpart PIG-1 (Figure S3 and S4), which would be related to the increased levels of ROS and the percentage of A375 cells in S phase. No significant differences in cyclin E, CDK2 and CDK4 levels were observed between catalase-treated and control cells and between non-tumor and tumor cells (Figure S3). Thus, the decrease in cyclin D1 levels observed would be involved in G1/S arrest induced by catalase in A375 cells.

### Catalase Treatment Induced Nuclear Localization of p27Kip1

**Figure 3:**
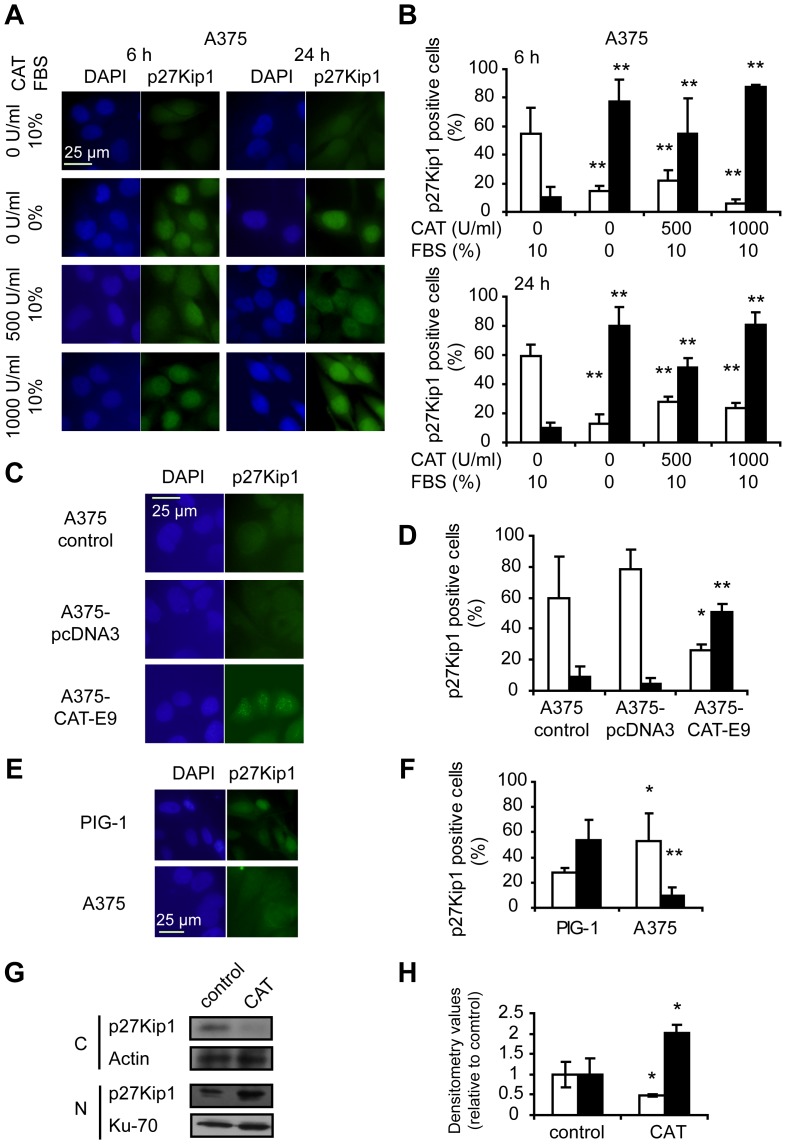
Nuclear localization of p27Kip1 in response to H_2_O_2_ scavenging and intrinsic low levels of H_2_O_2_. (A-F) Subcellular localization of p27Kip1 evaluated by immunocytofluorescence. (A–B) Melanoma cells treated with 500 and 1000 U/ml catalase (CAT) for periods of 6 or 24 h or left untreated. FBS starved cells were used as control of G1 arrest. (C–D) Catalase overexpression model (A375-CAT-E9 cells) vs. controls (A375-pcDNA3 and A375 control cells). (E-F) Non-tumor (PIG-1) vs. tumor (A375) cells. (A, C and E) Representative images of p27Kip1 immunocytofluorescence showing the subcellular localization of the protein. DAPI: staining of nuclear DNA; p27Kip1: FITC staining of p27Kip1 protein. (B, D and F) Percentage of positive (□) cytoplasms and positive (■) nuclei for p27Kip1 relative to the total number of counted cells. Data are expressed as mean ± SD. (B) **p<0.01 vs. control. (D) *p<0.05 and **p<0.01 vs. A375 control. (F) *p<0.05 and **p<0.01 vs. non-tumor cells. (G–H) Increased expression of nuclear p27Kip1 in A375 cells after 1000 U/ml catalase (CAT) treatment as compared with control A375 cells (treated with 1000 U/ml heat-inactivated catalase, IN-CAT) for 24 h, detected by western blot of nuclear and cytosolic extracts (see Methods). (G) Representative immunoblot images are shown. C: Cytoplasmic extracts; N: Nuclear extracts. Actin and Ku-70 densitometric values were used to standardize for cytoplasmic and nuclear protein loading, respectively. (H) Relative densitometric values of (□) cytoplasmic and (■) nuclear p27Kip1 levels. Results are referred to control cells. Data are expressed as mean ± SD. *p<0.05 vs. control.

Considering the G1 arrest induced by catalase and the importance of the subcellular localization of the inhibitory protein p27Kip1 for its regulatory activity, the effect of H_2_O_2_ scavenging on the localization of this protein was studied by immunofluorescence. Remarkably, p27Kip1 was localized primarily within the nucleus in melanoma cells treated with or overexpressing catalase as compared with controls, in which p27Kip1 distribution was predominantly cytoplasmic (Figures 3A–3D). In addition, melanocytes exhibited a higher percentage of positive p27Kip1 cells than that of A375 melanoma cells (Figures 3E and 3F). This protein was mainly localized in the nucleus in non-tumor cells and in the cytoplasm in tumor cells (Figures 3E and 3F).

In order to confirm the effects of H_2_O_2_ scavenging on p27Kip1 localization observed by immunofluorescence, the levels of this protein in nuclear and cytosolic extracts of melanoma cells treated with catalase were evaluated by western blot. We demonstrated a significant increase of p27Kip1 levels in nuclear extracts of cells treated with catalase as compared with control (Figure 3G and 3H).

These results demonstrate the modulation of the intracellular localization of p27Kip1 in the regulation of cell proliferation by catalase and confirm our previous findings [35], extending those results to human A375 cells. The persistence of p27Kip1 in the nucleus induced by H_2_O_2_ removal would favor the blockage of cell cycle at G1/S transition.

### H_2_O_2_ Modulation Leads to Post-translational Modifications of p27Kip1

**Figure 4:**
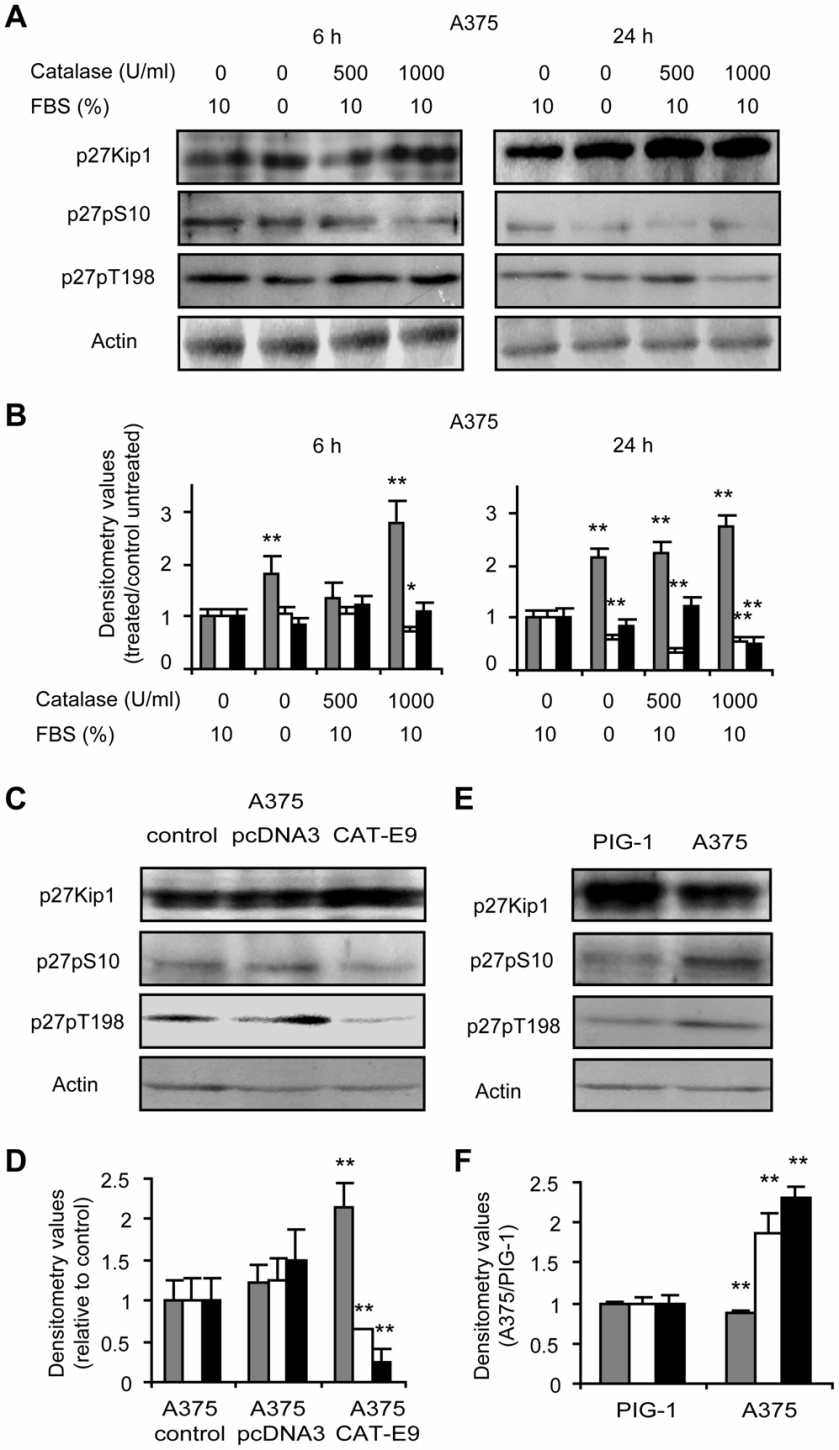
Increased p27Kip1 and decreased phosphorylated p27Kip1 at S10 and T198 by lowering H_2_O_2_ levels. The expression of p27Kip1 and p27Kip1 phosphorylated at S10 (p27pS10) and T198 (p27pT198) was analyzed by western blot. (A–B) A375 melanoma cells treated with catalase (CAT) for 6 and 24 h. FBS starved cells were used as control of G1 arrest. (C–D) Catalase overexpression model (A375-CAT-E9 cells) vs. controls (A375-pcDNA3 and A375 control cells). (E–F) Non-tumor (PIG-1) vs. tumor (A375) cells. (A, C and E) Representative immunoblot images. (B, D and F) Relative densitometric values of (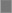) p27Kip1 levels, (□) p27pS10 and (■) p27pT198. Actin densitometric values were used to standardize for protein loading. Results are referred to control without treatment (in B and D) and to non-tumor (PIG-1) cells (in F). Data are expressed as mean ± SD. (B) *p<0.05 and **p<0.01 vs. untreated control. (D) **p<0.01 vs. A375 control. (F) **p<0.01 vs. non-tumor cells.

We also demonstrated a significant increase in the total levels of p27Kip1 in response to catalase treatment or overexpression assessed by western blot (Figure 4). This could be related to the high levels of p27Kip1 observed in the nucleus of catalase treated cells (Figure 3E). Moreover melanoma cells exhibited lower levels of this inhibitory protein as compared to melanocytes (Figure 4). Regarding western blot (Figure 4) and immunofluorescence (Figure 3) results and considering that p27Kip1 levels, function and localization are regulated by phosphorylations, the levels of p27Kip1 phosphorylated at S10 (p27pS10), T198 (p27pT198) and T187 (p27pT187) in response to H_2_O_2_ scavenging were evaluated by western blot. The phosphorylation of p27Kip1 at S10 and T198 is a key event for nuclear exportation of this protein and progression of cell cycle and we demonstrated a significant decrease in the levels of p27pS10 and p27pT198 in cells overexpressing or treated with catalase as compared with controls (Figure 4). Furthermore, PIG-1 melanocytes revealed lower levels of p27pS10 and p27pT198 than their tumor counterpart (Figure 4).

These findings suggest that reduced levels of H_2_O_2_ by catalase prevent the phosphorylation of specific sites of p27Kip1 therefore avoiding the nuclear exportation of the protein and leading to cell cycle arrest through the accumulation of p27Kip1 in the nucleus. In addition, the phosphorylation of p27Kip1 at T187, which is involved in triggering proteolysis of this protein, was evaluated in catalase-treated melanoma cells and no significant differences were observed vs. untreated controls (Figure S5).

**Figure 5:**
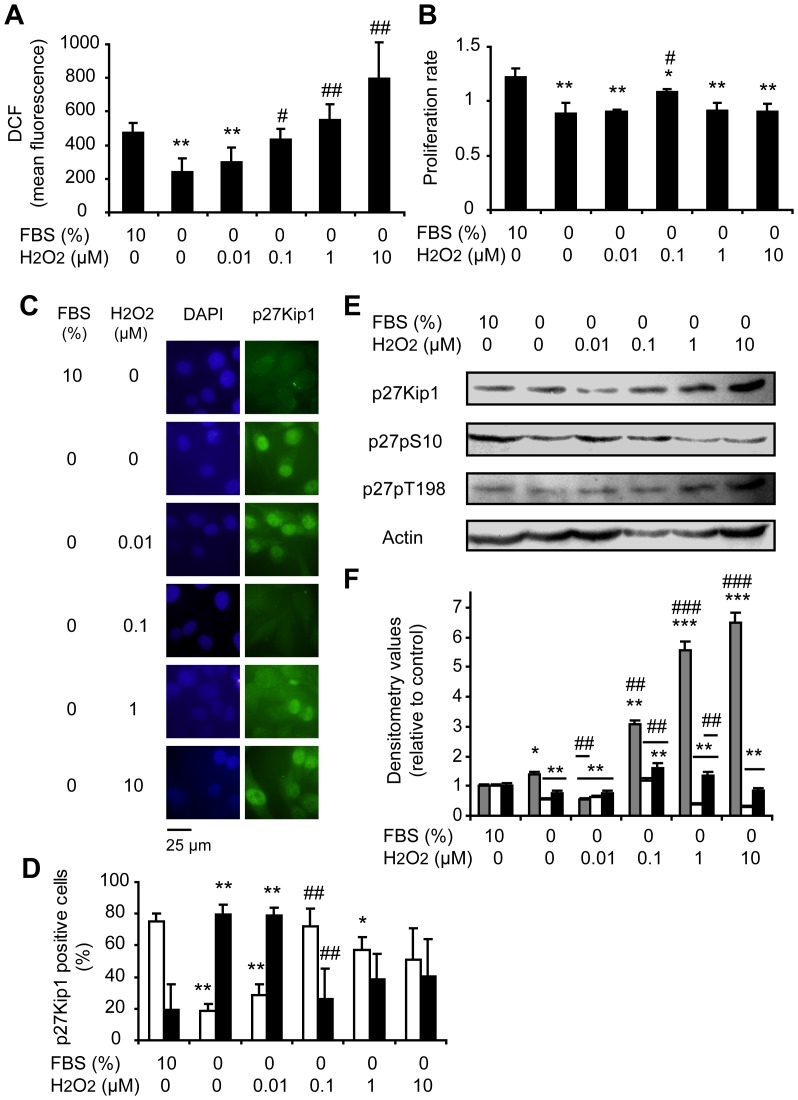
Adding 0.1 µM H_2_O_2_ to FBS starved cells regulates p27Kip1 phosphorylation and localization, favoring proliferation. Melanoma (A375) cells grown in complete medium with 10% FBS were arrested by FBS starvation (0% FBS) for a period of 24 h and then cells were incubated with different concentrations of H_2_O_2_ (0.01–10 µM) or to 10% FBS. (A) Intracellular ROS levels measured by DCFH-DA assay. (B) Cell proliferation rate evaluated by the MTT assay. (C) Representative images of p27Kip1 immunocytofluorescence showing the subcellular localization of the protein. DAPI: staining of nuclear DNA; p27Kip1: FITC staining of p27Kip1 protein. (D) Percentage of positive (□) cytoplasms and positive (■) nuclei for p27Kip1 relative to the total number of counted cells. (E) The expression of p27Kip1, p27pS10 and p27pT198 analyzed by western blot. (F) Relative densitometric values of (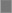) p27Kip1 levels, (□) p27pS10 and (■) p27pT198. Actin densitometric values were used to standardize for protein loading. Results are referred to control incubated with 10% FBS. (A, B, D and F) Data are expressed as mean ± SD. *p<0.05, **p<0.01 and ***p<0.001 vs. cells incubated with 10% FBS; ^#^p<0.05, ^##^p<0.01 and ^###^p<0.001 vs. FBS-starved cells not-exposed to H_2_O_2_.

Considering that growth factors trigger H_2_O_2_ production that leads to activation of signaling pathways governing cellular proliferation [27], we evaluated how H_2_O_2_ is involved in the modulation of p27Kip1 in G1-arrested A375 cells by FBS starvation incubated with different levels of H_2_O_2_ for 24 h. Figure 5A shows increased intracellular levels of ROS in a dose-dependent manner in cells treated with 0.1–10 µM H_2_O_2_ in comparison to FBS starved cells. It has been previously reported that the application of 0.1–7 µM H_2_O_2_ to cultured cells results in intracellular H_2_O_2_ levels of approximately 0.01–0.07 µM and directly stimulates cell proliferation. On the other hand, increasing amounts of cell death occur with applied concentrations of H_2_O_2_≥10 µM [reviewed in 26]. In our cellular model, incubation of FBS starved cells with 0.1 µM H_2_O_2_ induced an increase in proliferation rate in comparison to untreated FBS starved cells. On the other hand, no effect in cell proliferation was observed with the other doses of H_2_O_2_ used (Figure 5B). Cells treated with 0.1 µM H_2_O_2_ exhibited a predominantly cytoplasmic p27Kip1 distribution; similar to cells incubated with 10% FBS while p27Kip1 was found mainly in the nucleus in untreated FBS starved cells (Figures 5C and 5D). The subcellular localization of this protein in cells treated with 0.01 µM of H_2_O_2_ was comparable to the pattern observed in FBS starved cells and the addition of 1–10 µM of H_2_O_2_ to A375 FBS starved cells resulted in a similar percentage of nuclear and cytoplasmic p27Kip1 (Figures 5C and 5D). Western blots showed decreased levels of p27Kip1 in cells treated with 0.01 µM of H_2_O_2_ as compared to FBS starved cells and to cells treated with 0.1–10 µM of H_2_O_2_ (Figures 5E and 5F). The levels of p27pS10 and p27pT198 in cells treated with 0.1 µM of H_2_O_2_ increased as in cells incubated with 10% FBS while FBS starved cells showed low levels of p27pS10 and p27pT198 (Figures 5E and 5F). On the contrary, no significant differences were found in the levels of p27pT187 in cells treated with exogenous H_2_O_2_ (0.1 and 10 µM) as compared to both FBS starved and 10% FBS incubated control cells (Figure S5). These findings suggest that H_2_O_2_ at a mitogenic level of 0.1 µM for our cellular model regulates p27Kip1 phosphorylation leading to cytoplasmic localization of this protein and favoring cell proliferation.

Thus, we demonstrated that H_2_O_2_ would be implied in the modulation of key regulatory post-translational modifications of p27Kip1 protein in melanoma cells.

### Catalase also Modulates Cell Proliferation and Subcellular Localization of p27Kip1 in Colorectal Carcinoma and Neuroblastoma Cells

**Figure 6:**
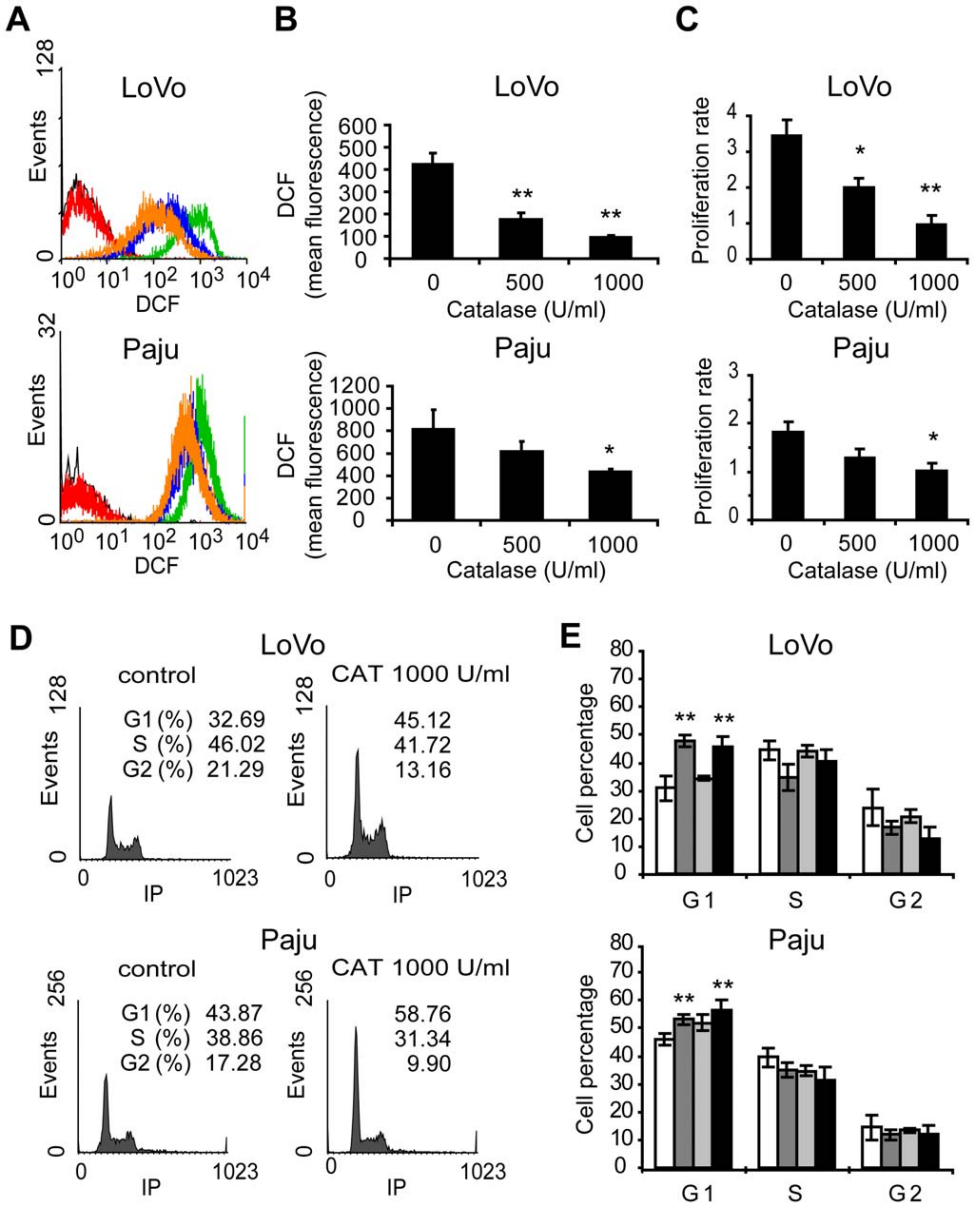
Colon adenocarcinoma and neuroblastoma cells show low proliferation rate and G1 arrest after catalase treatment. LoVo and Paju cells were treated with 0–1000 U/ml catalase (CAT) for 24 h. (A–B) Intracellular ROS levels determined by DCFH-DA assay. (A) Representative histograms of DCF fluorescence of cells treated with 500 (blue line) and 1000 (orange line) U/ml catalase or left untreated (green line) for 24 h. Control cells not exposed to DCFH-DA (black line) and control cells treated with catalase just before DCFH-DA incubation (red line). (B) DCF mean fluorescence (arbitrary units) vs. catalase dose. (C) Cell proliferation rate of LoVo and Paju cells treated with catalase for 24 h, relative to control cells, evaluated by the MTT assay. (D–E) Cell cycle analysis assessed by flow cytometry after staining with propidium iodide. (D) Representative histograms of DNA content cells treated with catalase (CAT). (E) Percentage of cells in the different phases of the cell cycle in response to CAT treatment. FBS starved cells were used as control of G1 arrest. (□) Untreated control cells, (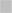) 500 U/ml and (■) 1000 U/ml CAT and (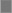) FBS starved cells. (B, C and E) Data are expressed as mean ± SD. *p<0.05 and **p<0.01 vs. control.

In order to extend the results observed for melanoma cells treated with catalase to other human cancer cell types, we evaluated cell proliferation, cell cycle and p27Kip1 intracellular distribution in colorectal carcinoma (LoVo) and neuroblastoma (Paju) cells. The characterization of our cellular models at ROS level showed that LoVo cells exhibited lower intracellular ROS levels than Paju and A375 cells (Figure S6). Interestingly, we observed a low proliferation rate (p<0.01) for both LoVo and Paju cells (Figure 6) in response to the reduced levels of ROS induced by the addition of catalase to cell cultures (Figure 6). LoVo and Paju cells treated with catalase for 24 h exhibited a significant G1 cell cycle arrest (Figure 6) associated with a decrease in cyclin D1 levels (Figure S7). In agreement with these results, the signal for cyclin D1 detected by immunofluorescence was extremely low in the nucleus of cells treated with catalase and a significant decrease of the percentage of positive nuclei was found in these cells as compared with control cells (Figure S8). No significant differences in cyclin E, CDK2 and CDK4 levels were observed between catalase-treated and non-treated cells (Figure S7).

**Figure 7:**
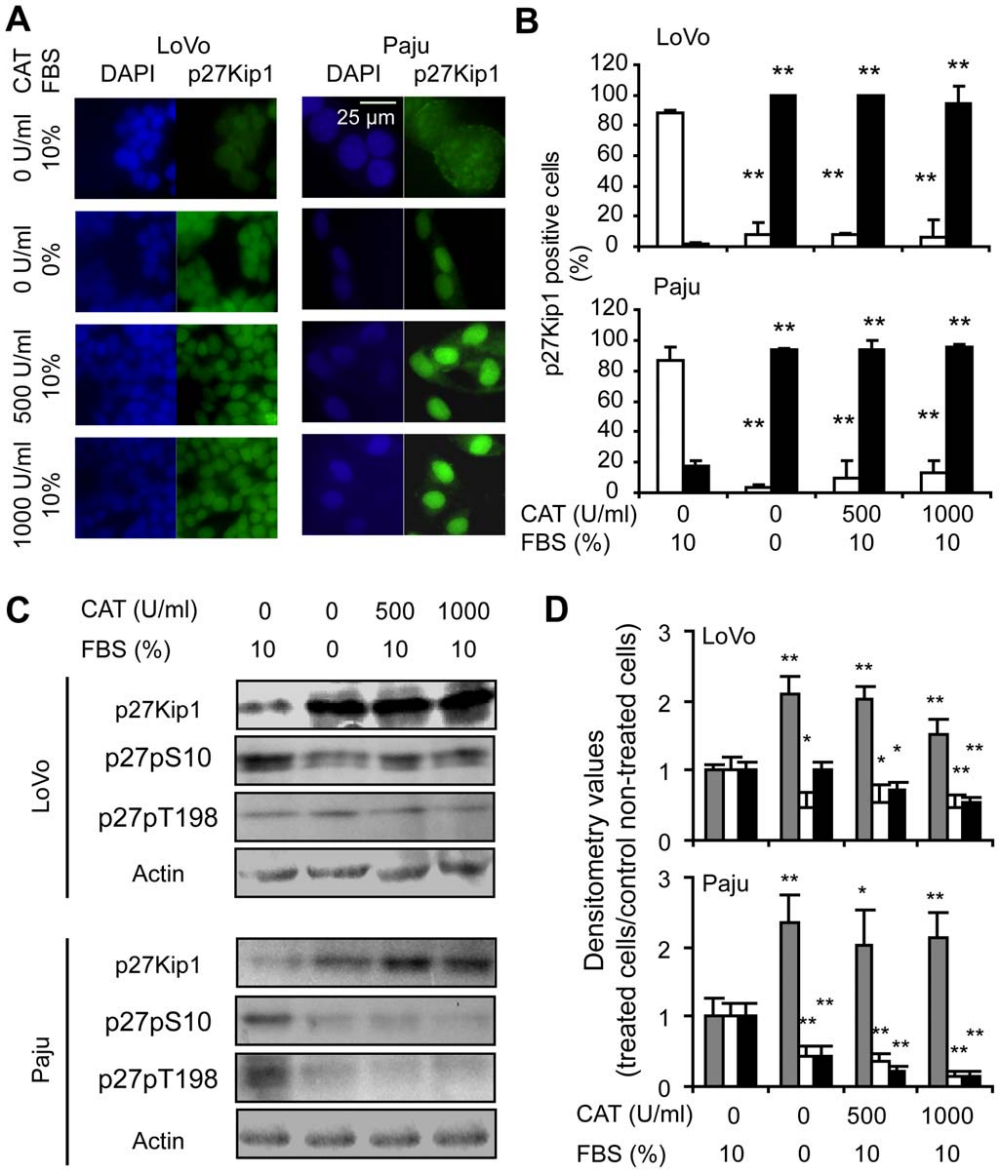
Relocalization of p27Kip1 in colon adenocarcinoma and neuroblastoma cells after 24 h of catalase treatment. (A and B) Nuclear localization of p27Kip1 with catalase (CAT) was detected by immunocytofluorescence. (A) Representative images of p27Kip1 immunocytofluorescence showing the subcellular localization of the protein. DAPI: staining of nuclear DNA; p27Kip1: FITC staining of p27Kip1 protein. (B) Percentage of positive cytoplasms (□) and positive nuclei (■) for p27Kip1 relative to the total number of counted cells. (C and D) Increase of p27Kip1 levels and decrease of p27Kip1 phosphorylated at S10 (p27pS10) and T198 (p27pT198) in response to H_2_O_2_ scavenging, analyzed by western blot. (C) Representative immunoblot images. (D) Relative densitometric values of (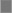) p27Kip1 levels, (□) p27pS10 and (■) p27pT198. Actin densitometric values were used to standardize for protein loading. Results are referred to control without treatment. (B and D) Data are expressed as mean ± SD. *p<0.05 and **p<0.01 vs. untreated control. (A–D) FBS starved cells were used as control of G1 arrest.

Colorectal carcinoma and neuroblastoma cells treated with catalase also showed p27Kip1 localized primarily within the nucleus as compared with controls, in which p27Kip1 distribution was predominantly cytoplasmic (Figures 7A and 7B show treatments for 24 h and Figures S9A and S9B show treatments for 6h). In addition, we demonstrated by western blot a significant increase in the levels of p27Kip1 in response to catalase treatment for both LoVo and Paju cells (Figures 7C and 7D treatments for 24 h and Figures S9C and S9D treatments for 6 h). Finally, we reproduced a significant decrease in the levels of p27pS10 and p27pT198 in colorectal carcinoma and neuroblastoma cells treated with catalase as compared with controls (Figures 7C and 7D treatments for 24 h and Figures S9C and S9D treatments for 6 h).

These results confirmed and extended our previous findings. We suggest that ROS decrease in different human cancer cells by catalase regulates the subcellular localization of p27Kip1 avoiding the phosphorylation of the protein at key sites (S10 and T198) leading to the accumulation of p27Kip1 in the nucleus which favors cell cycle arrest.

## Discussion

In this study, we demonstrated a modification in the subcellular localization of p27Kip1 mediated by changes in the phosphorylation of specific residues of this protein in response to H_2_O_2_ level variations. The nuclear increase of p27Kip1 in melanoma cells either overexpressing or treated with catalase was associated with a decrease in the levels of the phosphorylated protein at S10 and T198. These findings, combined with the decrease in cyclin D1 levels induced by H_2_O_2_ scavenging, would favor the cell cycle arrest at G1 phase and the inhibition of cell proliferation. We also demonstrated that the addition of H_2_O_2_ in a dose that induces proliferation to FBS starved cells increases the levels of p27pS10 and p27pT198 leading to cytoplasmic localization of this protein. The other human cancer cells, derived from colorectal carcinoma and neuroblastoma, used in our study, showed a similar response to catalase treatment, extending the results observed for melanoma cells. In addition, melanocytes, which exhibited low levels of ROS as compared with melanoma cells, showed decreased levels of p27pS10 and p27pT198 and nuclear localization of the protein in association with a low proliferation rate.

The difference in intracellular ROS levels observed among the tumor cell lines studied herein agrees with a previous work by Szatrowski et al [40] that reported higher levels of H_2_O_2_ in several melanoma, neuroblastoma, ovarian carcinoma cell lines and one type of colon carcinoma cells compared to other colon carcinoma cell line and breast and pancreatic carcinoma cells, which showed similar H_2_O_2_ levels to those of normal cells. Considering the increased levels of ROS observed in A375 melanoma cells as compared to PIG-1 melanocytes, we generated a model of catalase overexpression with A375 cells. The presence of higher levels of ROS in tumor cells than their non-tumor counterpart observed herein is in agreement with other reports [23]–[24], [42]–[45]. In a previous report [23], we demonstrated that H_2_O_2_ scavenging resulted in a significant inhibition of cell proliferation in tumor cells of different origin [23] and we showed herein that catalase treatment or overexpression induced an arrest in the G1 phase of the cell cycle. Our results are consistent with the association between G1 arrest and decreased ROS levels reported in other experimental conditions [35], [46]–[49].

Growth factors trigger H_2_O_2_ production that leads to activation of signaling pathways governing cellular proliferation, such as mitogen-activated protein kinases (MAPKs) [27]. This could explain the decrease in ROS levels observed in FBS starved cells in our melanoma model. Previously, we demonstrated decreased ROS levels in FBS starved squamous carcinoma cells [35]. We also observed that 0.1 µM of H_2_O_2_ added to FBS starved A375 cells induced cell proliferation and it has been previously reported [26] that this dose results in a 10 nanomolar intracellular concentration of H_2_O_2_ and directly stimulates cell proliferation. On the contrary, the addition of both lower and higher H_2_O_2_ concentrations did not produce changes in cell proliferation. Stone and Yang [26] reviewed that doses around or higher than 10 µM of H_2_O_2_ applied to cell cultures are associated with cell death increase or, at least, with initially growth arrest, which may be followed by growth promoting adaptation to oxidative stress. They also reported that 0.01 µM of H_2_O_2_ added to cell cultures results in intracellular H_2_O_2_ levels of ∼1 nM which are extremely low to detect any cellular response [26]. However, the precise transition points for cellular responses to oxidative stress may vary due to cell type and culture conditions [26]. A375 cells exhibited intrinsic high levels of ROS combined with increased proliferation rate; perhaps those levels of ROS were bordering cytotoxicity and the addition of 1–10 µM of H_2_O_2_ induced the activation of other signaling pathways related to stress response. Intracellular levels of ROS become critical when cells are committed to proliferate [50]. In this sense, in our melanoma model we showed the inhibition of cell growth by decreasing the physiological levels required to induce signals to proliferate and on the other hand by adding supraphysiological levels of H_2_O_2_ that would induce stress signals.

It has been previously demonstrated that ROS are involved in promoting mitogenesis by modulating cyclin D1 levels [33]. In agreement with Brar et al [41], in our cellular models, we found a decrease in the protein levels of cyclin D1 associated with the inhibition of proliferation induced by decreasing ROS levels with catalase, which could be related to the modulation of ERK1/2 activity [47], [48]. Moreover, we found low nuclear signal of cyclin D1 and a significant diminution of the percentage of positive nuclei for this protein in response to H_2_O_2_ scavenging revealed by immunofluorescence, suggesting that ROS levels diminution by catalase would favor cyclin D1 degradation. On the other hand, we have not observed differences in cyclin E, CDK2 and CDK4 levels between catalase-treated and non-treated cells and between non-tumor and tumor cells.

Considering p27Kip1, it plays a crucial role in cell cycle regulation by virtue of its ability to respond to modifications in the growth environment of the cell, integrating diverse signals into a final decision between proliferation and cell cycle exit [2], [3]. This protein remains in the nucleus in quiescent cells, but it is exported to the cytoplasm in response to proliferating signals [3], where it can be degraded or stabilized to be involved in the regulation of other processes such as cell migration [4]. In the present study, we demonstrated a modulation on the levels of this regulatory protein and a differential intracellular localization depending on ROS levels. The scavenging of H_2_O_2_ by catalase induced an increase in the levels of p27Kip1 and the nuclear localization of the protein, while control proliferating cells showed mainly cytoplasmic localization of this protein. Moreover, we demonstrated that p27Kip1 exhibited a predominantly cytoplasmic distribution in FBS starved melanoma cells exposed to a proliferating dose of H_2_O_2_ (0.1 µM). It has been reported that the oncogenic activation of RTK, PI3K, SRC, or Ras-MAPK pathways cooperate to inactivate p27Kip1, accelerate its proteolysis or change its intracellular localization in human cancers through modifications in p27Kip1 phosphorylation [5]. Thus, considering that H_2_O_2_ has been described as mediator of RTK/Ras, MAPKs, PI3K/AKT and non-receptor tyrosine kinases pathways [51], cell treatment with H_2_O_2_ in a proliferating dose would induce the nuclear export or degradation of p27Kip1 while the scavenging of H_2_O_2_ would be preventing these effects maintaining this protein at the nucleus.

Regarding p27Kip1 phosphorylations, this protein may be phosphorylated at multiple sites [3]. Most of these post-translational modifications are on threonine and serine residues [9] and phosphorylations on tyrosine residues have recently been reported [16], [17]. Taking into account that we demonstrated changes in the subcellular distribution of p27Kip1 in response to H_2_O_2_ modulation, we studied the levels of p27pS10 and p27pT198 because phosphorylation of p27Kip1 at those sites in proliferating cells, enables its nuclear exportation [6], [8], [9], leading to accumulation of p27pT198 in the cytoplasm [4], [9]. We demonstrated a decrease in the levels of p27pS10 and p27pT198 in response to H_2_O_2_ removal in tumor cells of different origin. In addition, melanocytes exhibited lower levels of p27Kip1 phosphorylated at those sites than their tumor counterpart. Other authors reported the involvement of AKT and p90 ribosomal S6 kinase (RSK1) in the phosphorylation of p27Kip1 at T198 [9], [52] and AKT and human kinase interacting stathmin (hKIS) in the phosphorylation of the protein at S10 [53], [54]. Interestingly, all of these kinases take part in signaling pathways regulated by ROS [30], [51], [55]. Thus, these data suggest that H_2_O_2_ blockage would avoid nuclear exportation of p27Kip1 by modulating the phosphorylation of specific sites, leading to the cell cycle arrest through the accumulation of this protein in the nucleus. Moreover, preliminary results of our laboratory showed a decrease in AKT1 and hKIS gene expression in A375 cells treated with catalase in comparison to non-treated cells (unpublished data), which could be associated to the decrease on p27pS10 and p27pT198. On the other hand, the addition of H_2_O_2_ to FBS starved cells at a proliferating dose of 0.1 µM led to an increase in p27pS10 and p27pT198 levels associated to a cytoplasmic distribution of p27Kip1 and these findings confirmed the involvement of H_2_O_2_ in the modulation of key regulatory post-translational modifications of p27Kip1 protein. Subcellular distribution, levels and phosphorylation status of p27Kip1 in cells treated with higher doses of exogenous H_2_O_2_ than 0.1 µM might suggest that these concentrations in our melanoma model could be implied in other cellular processes related to the oxidative stress response.

Unlike other well characterized tumor suppressors, p27Kip1 is rarely mutated or deleted in human cancers [5], [9]. Rather it is frequently deregulated: p27Kip1 protein levels are reduced (due to accelerated proteolysis or impaired translation) or the protein suffers sequestration in cyclin D-CDK complexes or is mislocalized to the cytoplasm [5], [9]. Cytoplasmic p27Kip1 was associated to invasive and metastatic tumors [56], [57]. Phosphorylations of p27Kip1 at S10 and T198 play a decisive role in the nuclear export of the protein and in its permanency in the cytoplasm where p27Kip1 would perform other activities, such as those related to cell motility [4], [57]. Interestingly, in this study, we found an increase in p27pT198 levels in melanoma cells treated with high doses of H_2_O_2_ (1–10 µM) and this phosphorylation site is involved in stabilization of the protein at the cytoplasm, while the increase in p27pS10 was only found for the proliferating dose of H_2_O_2_. These findings suggest that p27pS10 rather than p27pT198 would be mainly involved in the proliferating effect of 0.1 µM H_2_O_2_ in our melanoma model. In this regard, Schiappacassi et al [58] recently showed that p27Kip1 phosphorylation at T198 does not affect cell proliferation while this event is important in cell motility regulation. The phosphorylation of this protein on T187 by cyclin E-CDK2 complex targets p27Kip1 for ubiquitin-dependent proteolysis [9]–[11]. In our melanoma cellular model, non significant differences were observed in p27pT187 levels after ROS modulation, both by addition or scavenging of H_2_O_2_, which is consistent with the fact that no variations were observed on cyclin E and CDK2 levels after catalase treatment. This suggests that the mechanisms that control p27Kip1 levels by proteolysis are not affected by ROS levels and our findings support the hypothesis that the pro-oxidant levels of melanoma cells allow the nuclear exportation and stabilization of p27Kip1 in the cytoplasm by its phosphorylation on S10 and T198 due to ROS-regulated signaling pathways, such as, PI3K/AKT pathway.

Considering our results in relation to the fact that most cancer cells exhibit high levels of ROS, we suggest another mechanism by which cancer cells, such as the types studied herein, taking advantage of their intrinsic ROS levels would favor cell proliferation and malignant features, altering p27Kip1 subcellular localization through an increase in the levels of p27pS10 and p27pT198. These phosphorylations lead to cytoplasmic localization and stabilization of the protein which, in turn, has been associated with increased malignancy. In this sense, Bottini et al. demonstrated a cytoplasmic accumulation of p27Kip1 in colorectal cancer specimens of patients with poor outcomes for cancer-related relapse and survival [59]. A recent study of tissue microarrays of human melanocytic lesions by Chen et al. also revealed that nuclear p27Kip1 expression was reduced in primary melanomas compared with dysplastic nevi and further reduced in metastatic melanoma, whereas the cytoplasmic p27Kip1 was increased in primary and metastatic melanomas compared with dysplastic nevi [18].

Our study of both nuclear levels and cytoplasmic mislocalization of p27Kip1 by H_2_O_2_ modulation contributes in some manner to the understanding of the potential prognostic and predictive value of the protein, as it was recently noted by Wender et al [57], since reduced nuclear p27Kip1 increases proliferation, and cytoplasmic p27Kip1 would drive tumor cell invasion.

## Materials and Methods

### Cell Lines and Culture

The following human cell lines were used: PIG-1 [60], A375 [44], [61], LoVo [44] and Paju [62], [63]. PIG-1 melanocytes and melanoma A375 cells were kindly provided by Dr. I.C. Le Poole (Departments of Pathology, Microbiology and Immunology, Oncology Institute, Loyola University, Maywood, Illinois, USA) and Dr. E. Medrano (Huffington Center on Aging, Departments of Molecular & Cellular Biology and Dermatology, Baylor College of Medicine, Houston, Texas, USA) respectively. Colorectal carcinoma LoVo cells (CCL-229) were kindly donated by Dr. O. Podhajcer (Laboratorio de Terapia Celular y Molecular, Fundación Instituto Leloir, Buenos Aires, Argentina). Neuroblastoma Paju cells were provided by Dr. E. Rivera (Laboratorio de Radioisótopos, Facultad de Farmacia y Bioquímica, Universidad de Buenos Aires, Argentina). PIG-1 cells were grown in 254 medium (Cascade Biologics) supplemented with Human Melanocyte Growth Supplement (HMGS, Cascade Biologics). A375 and LoVo cells were maintained in 50∶50 of DMEM/Ham’s F12 (Invitrogen, Argentina). A375 medium was also supplemented with 17.6 µg/ml ascorbic acid (Sigma), 150 µg/ml pyruvic acid (Sigma), 300 µg/ml galactose (Sigma) and 5 µg/ml insulin. Paju cells were maintained in RPMI-1640 (Invitrogen, Argentina). All media were supplemented with 50 U/ml penicillin, 50 µg/ml streptomycin and 10% (v/v) FBS (NatoCor, Córdoba, Argentina) and cells were grown at 37°C in a 5% CO_2_ humidified atmosphere. Cells were regularly tested to be mycoplasma-free.

### Treatments and Generation of a Catalase-overexpression Model

For H_2_O_2_ scavenging experiments, cells were incubated with 0–1000 U/ml catalase (Sigma) added to complete culture medium for periods of 6 or 24 h. A solution of catalase in phosphate buffered saline (PBS) sterilized by filtration was prepared fresh just before addition to the medium. Control cells were non-treated or treated with a solution of 1000 U/ml of heat-inactivated catalase in PBS. In order to obtain a catalase-overexpression model, A375 cells were stably transfected with a construct containing the pcDNA3 expression vector and the cDNA coding for human catalase (CAT-pcDNA3), using Lipofectamine 2000 (Invitrogen, Argentina) as previously described [23]. Control cells were transfected with empty pcDNA3 vector. For selection of stable transfectants, geneticin (1000 µg/ml, Sigma) was added to the cell medium 24 h after transfection and maintained for 3 weeks changing the medium with geneticin every two days. Geneticin-resistant clones were obtained by dilution cloning.

For experiments with exogenous H_2_O_2_, after 24 h of FBS starvation, cells were incubated with rising concentrations of H_2_O_2_ (0.01–10 µM) or 10% of FBS added to the medium for a period of 24 h.

### Determination of ROS Levels

In order to validate and characterize our cellular models of H_2_O_2_ scavenging, both by exogenous treatments with catalase and by overexpression of this enzyme, the levels of ROS, the expression (Methods S1) and the activity of catalase (Methods S1) were determined.

The levels of intracellular ROS were determined by 2′, 7′-dichlorodihydro-fluorescein diacetate (DCFH-DA, Molecular Probes) assay as previously described [35]. Briefly, cells treated with catalase for 24 h or stably transfected with CAT-pcDNA3 or transfected with the empty vector or left untreated (controls) were washed twice with PBS and incubated with 10 µM DCFH-DA in PBS at 37°C for 30 min, protected from light. After incubation, cells were washed with PBS, harvested with trypsin/EDTA and evaluated by flow cytometry (FACSCalibur, Becton Dickinson). Ten thousand cells were measured for each experimental condition. The DCFH-DA assay is widely used for the measurement of H_2_O_2_ levels but other intracellular ROS can oxidize the probe, and in order to appraise the specificity of H_2_O_2_ determination by this technique, control cells were treated with 1000 U/ml catalase throughout the assay, added just before DCFH-DA incubation [35]. Data were analyzed with WinMDI software. Three experiments were performed with triplicates per each experimental condition. This DCFH-DA assay was also used to determine ROS levels in FBS starved cells treated with rising concentrations of H_2_O_2_ or 10% FBS added to the medium for a period of 24 h.

### Cell Growth and Cell Cycle Analysis

The 3-(4,5-dimethylthiazol-2-y1)-2,5-diphenyltetrazolium bromide (MTT) growth assay [64] was performed at 24 h post-treatment in cells of our models of H_2_O_2_ scavenging (treated with catalase or transfected with CAT-pcDNA3) growing in 24-well plates as previously described [23], [35]. Control cells were left untreated, transfected with empy vector or incubated with heat-inactivated catalase. Results were expressed as proliferation rate. All experiments were performed at least three times with quadruplicate measurement per condition.

Cell cycle analysis was performed by propidium iodide (PI) staining. Subconfluent cells with or without catalase treatment for 24 h or transfected with CAT-pcDNA3 or empty vector were trypsinized, collected by centrifugation, and washed with ice-cold PBS before fixing in 96% ethanol at 4°C. Fixed cells were resuspended in 0.2 ml PBS containing 50 µg/ml RNase I (Sigma) and 60 µg/ml PI (Sigma). FBS starved cells were used as control of G1 arrest. The number of cells in the different phases of the cell cycle was determined by flow cytometry (FACSCalibur, Becton Dickinson). Ten thousand cells were measured per experimental condition and analyzed with WinMDI and Cylchred software. Three experiments were performed with triplicates per experimental condition.

### Detection of p27Kip1 by Immunocytofluorescence

Subconfluent cell cultures grown in 60 mm dishes were fixed in 4% (w/v) paraformaldehyde in PBS for 15 min. Cells were then washed with PBS, permeabilized with 0.5% (v/v) Triton X-100 in PBS for 15 min, washed and blocked with 5% (v/v) FBS in PBS for 30 min. Cells were incubated overnight at 4°C with the polyclonal anti-p27Kip1 (M-197, Santa Cruz Biotechnology) antibody, 1∶300 in PBS, washed and incubated with secondary FITC-conjugated anti-rabbit IgG (Sigma) for 1 h in the dark at room temperature. Finally, the samples were washed, counterstained and mounted with 1 µg/ml 4′,6-diamidine-2′-phenylindole (DAPI, Sigma) in an antifade solution in the dark. Cells were examined in an Olympus BX51 epifluorescence microscope utilizing immersion oil with a 100X (UPlanApo 100 X/1.35 oil) objective lens. For each treatment condition, FITC and DAPI images were serially captured by a CCD camera (Olympus DP70) and more than 50 fields containing approximately 20 cells each were stored. A code number was given to each image. Random sampling methods were used to select the images and all the cells in each selected image were screened. An average of 250 cells was evaluated per experimental condition. Total cells, positive cells, positive cytoplasms and positive nuclei for p27Kip1 were counted by eye by two scorers and results were crosschecked. FBS starved cells were used as control of G1 arrest. Three independent experiments were performed with triplicates per condition.

This method was used in a similar way to detect cyclin D1 by immunofluorescence (Methods S2).

### Determination of p27Kip1 and Phosphorylated p27Kip1 by Western Blot

Cells were treated with catalase or H_2_O_2_ or left untreated for 6 or 24 h. In the catalase-overexpression model cells were transfected with the CAT-pcDNA3 or with the empty vector. Cells incubated with heat-inactivated catalase were also used as a negative control. FBS starved cells were used as control of G1 arrest. In order to obtain the whole protein extract and the cytoplasmic and nuclear protein fractions, cells were washed twice and scraped in 1 ml PBS. A 0.2 ml aliquot was centrifuged and cells were incubated on ice for 30 min in RIPA lysis buffer (Sigma) containing the Halt protease and phosphatase inhibitor cocktail (Thermo Scientific) for the whole extract. The remaining aliquot was centrifuged and cells were lysed in 70 µl of extraction buffer (10 mM Hepes, 0.2 M sucrose, 15 mM KCl, 2 mM EDTA, pH 7.6). After 10 min of 2200 rpm centrifugation, the supernatant containing cytoplasmic proteins was collected. In order to obtain the nuclear proteins, the pellet was resuspended in 30 µl of extraction buffer with 5% glycerol and left 40 min at 4°C, vortexing every 5 min. After 10 min of centrifugation at 12000 rpm, the nuclear proteins were collected from the supernatant.

The protein yield was quantified by the DC Protein Assay Reagent (BioRad) based on the Lowry protocol. Samples were separated by SDS polyacrylamide (Promega) gel electrophoresis, transferred to nitrocellulose membranes (Hybond ECL Membrane, Amersham Biosciences, GE Healthcare) and immunoblotted by appropriate antibodies.

The antibodies against p27Kip1 (M-197), phosphorylated p27Kip1 protein at serine 10 and threonine 198: p27pS10 (Ser 10-R), p27p198 (Thr 198), Ku-70 (G-7) and actin (I-19) were purchased from Santa Cruz Biotechnology. The primary antibodies were detected using horseradish peroxidase-linked donkey anti-rabbit IgG (Amersham, GE Healthcare) or anti-goat IgG (Santa Cruz Biotechnology) and visualized by the ECL detection system (Amersham Biosciences, GE Healthcare). Quantification was performed by densitometric scanning with the NIH Image J software. Actin densitometric values were used to standardize for both the whole and cytoplasmic protein extracts loading and Ku-70 densitometric values were used to standardize for nuclear protein loading. Three independent experiments were performed with duplicates per experimental condition.

The detection of the other G1/S regulatory proteins and p27Kip1 phosphorylated at T187 by western blot is described in Methods S3.

### Statistical Analysis

Data are presented as mean ± SD. Significant changes were assessed using two-tailed Student’s t-test to compare two sets of data and one-way analysis of variance to compare three or more sets of data followed by Tukey’s multiple comparisons test to determine significant differences between group means. P-values less than 0.05 were considered significant for all tests.

## Supporting Information

Figure S1 **Characterization of the catalase-overexpression model.** (A) Clone A375-CAT-E9 showed the lowest intracellular ROS levels of the stable geneticin-resistant clones generated. DCF mean fluorescence (arbitrary units) of A375 cells stably transfected with a construct containing the pcDNA3 expression vector and the cDNA coding for human catalase (A375-CAT). Control cells were either transfected with empty pcDNA3 vector (A375-pcDNA3) or left non-transfected (A375 control). (B) Increased levels of catalase in clone A375-CAT-E9 as compared with A375-pcDNA3 or A375 control, determined by western blot. (C) Higher catalase activity of A375-CAT-E9 cells than control ones (A375-pcDNA3 or A375 control). (A and C) Data are expressed as mean ± SD. **p<0.01 vs. A375 control.
(TIF)

Figure S2 **Cells treated with heat-inactivated catalase exhibited no significant differences with non treated cells.** (A) The levels of ROS were measured by the DCFH-DA assay and (B) the proliferation rate by the MTT assay. A375 melanoma cells were treated with 1000 U/ml of catalase (CAT) or 1000 U/ml heat-inactivated catalase (IN-CAT) in PBS for 24 h or left untreated (control). Data are expressed as mean ± SD. *p<0.05 and **p<0.01 vs. A375 control.
(TIF)

Figure S3 **Cyclin D1 levels decreased in response to H_2_O_2_ scavenging and intrinsic low levels of H_2_O_2_.** The expression of cyclins and CDKs of G1/S was analyzed by western blot. (A and D) Melanoma cells treated with catalase (CAT) for 6 and 24 h. FBS starved cells were used as control of G1 arrest. (B and E) Catalase overexpression model (A375-CAT-E9 cells) vs. controls (A375-pcDNA3 and A375 control cells). (C and F) Non-tumor (PIG-1) vs. tumor (A375) cells. (A–C) Representative western blot images. (D–F) Relative densitometric values of cyclins and CDKs. Actin densitometric values were used to standardize for protein loading. Data are expressed as mean ± SD. (D) *p<0.05 and **p<0.01 vs. control untreated. (E) *p<0.05 vs. A375 control (F) **p<0.01 vs. non-tumor cells.
(TIF)

Figure S4 **Immunocytofluorescence confirmed the decrease in cyclin D1 in response to catalase treatment in melanoma cells.** Monoclonal anti-cyclin D1 (A-12, Santa Cruz Biotechnology) antibody, 1∶300 in PBS, and secondary FITC-conjugated anti-mouse IgG (Sigma) were used for immunocytofluorescence technique. (A–B) Melanoma cells treated with 500 and 1000 U/ml catalase (CAT) for periods of 6 or 24 h or left untreated. FBS starved cells were used as control of G1 arrest. (C–D) Catalase overexpression model (A375-CAT-E9 cells) vs. controls (A375-pcDNA3 and A375 control cells). (E–F) Non-tumor (PIG-1) vs. tumor (A375) cells. (A, C and E) Representative images of cyclin D1 immunocytofluorescence showing the subcellular localization of the protein. DAPI: staining of nuclear DNA; Cyclin D1: FITC staining of cyclin D1 protein. (B, D and F) Percentage of positive cells for cyclin D1 relative to the total number of counted cells. Data are expressed as mean ± SD. (B) **p<0.01 vs. untreated control. (D) **p<0.01 vs. A375 control. (F) **p<0.01 vs. non-tumor cells.
(TIF)

Figure S5 **Phosphorylation of p27Kip1 on T187 is not modulated by H_2_O_2_ in melanoma cells.** Melanoma (A375) cells grown in complete medium with 10% FBS were arrested by FBS starvation (0% FBS) for a period of 24 h or left untreated and then cells were incubated with different concentrations of H_2_O_2_ (0.1 or 10 µM) or to 10% FBS. Untreated cells were incubated with catalase 500 or 1000 U/ml. The expression of p27Kip1 and p27pT187 were analyzed by western blot. (A) Representative immunoblot images. (B) Relative densitometric values of p27pT187 referred to p27Kip1. Actin densitometric values were used to standardize for protein loading. Results are referred to control incubated with 10% FBS.
(TIF)

Figure S6
**Intracellular ROS levels in tumor cells of different origin determined by DCFH-DA assay.** Colorectal carcinoma cells (LoVo) exhibited lower intracellular ROS levels than neuroblastoma (Paju) and melanoma (A375) cells. (A) Representative histograms of DCF fluorescence: control cells not exposed to DCFH-DA (■), control cells treated with catalase just before DCFH-DA incubation (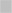) and cells incubated with DCFH-DA (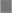). (B) DCF mean fluorescence (arbitrary units) of tumor cells. Data are expressed as mean ± SD. **p<0.01 vs. A375 cells.(TIF)

Figure S7 **Decrease of cyclin D1 by catalase was also found in colon adenocarcinoma and neuroblastoma cells.** The expression of cyclins and CDKs of G1/S was analyzed by western blot in (A and C) LoVo and (B and D) Paju cells treated with catalase (CAT) for 6 and 24 h. FBS starved cells were used as control of G1 arrest. (A and B) Representative western blot images. (C and D) Relative densitometric values of cyclins and CDKs. Actin densitometric values were used to standardize for protein loading. Data are expressed as mean ± SD. **p<0.01 vs. control untreated.
(TIF)

Figure S8 **Low signal of cyclin D1 after catalase treatment in LoVo and Paju cells by immunocytofluorescence.** See Methods S2 for immunocytofluorescence technique. (A) Representative images of cyclin D1 immunocytofluorescence showing the subcellular localization of the protein in tumor cells treated with 500 and 1000 U/ml catalase (CAT) for periods of 6 or 24 h compared to untreated controls. FBS starved cells were used as control of G1 arrest. DAPI: staining of nuclear DNA; Cyclin D1: FITC staining of cyclin D1 protein. (B) Percentage of positive cells for cyclin D1 relative to the total number of counted cells. Data are expressed as mean ± SD. **p<0.01 vs. untreated control.
(TIF)

Figure S9
**Relocalization of p27Kip1 in colon adenocarcinoma and neuroblastoma cells after 6 h of catalase treatment.** (A and B) Nuclear localization of p27Kip1 induced by catalase (CAT) was detected by immunocytofluorescence. (A) Representative images of p27Kip1 immunocytofluorescence showing the subcellular localization of the protein. DAPI: staining of nuclear DNA; p27Kip1: FITC staining of p27Kip1 protein. (B) Percentage of positive cytoplasms (□) and positive nuclei (■) for p27Kip1 relative to the total number of counted cells. (C and D) Increase of p27Kip1 levels and decrease of p27Kip1 phosphorylated at S10 (p27pS10) and T198 (p27pT198) in response to H_2_O_2_ scavenging, analyzed by western blot. (C) Representative immunoblot images. (D) Relative densitometric values of (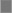) p27Kip1 levels, (□) p27pS10 and (■) p27pT198. Actin densitometric values were used to standardize for protein loading. Results are referred to control without treatment. (B and D) Data are expressed as mean ± SD. *p<0.05 and **p<0.01 vs. untreated control. (A–D) FBS starved cells were used as control of G1 arrest.(TIF)

Methods S1 **Catalase expression determination by western blot and measurement of catalase activity.**
(DOC)

Methods S2 **Detection of cyclin D1 by immunocytofluorescence.**
(DOC)

Methods S3 **Determination of G1/S regulatory proteins and p27Kip1 phosphorylated at T187 by western blot.**
(DOC)
