# Supplementary figures and images for: Phosphorylation and Subcellular Localization of p27Kip1 Regulated by Hydrogen Peroxide Modulation in Cancer Cells
Source: PLoS One. 2012 Sep 6;7(9):e44502. doi: 10.1371/journal.pone.0044502 (PMC3435274; doi:10.1371/journal.pone.0044502)

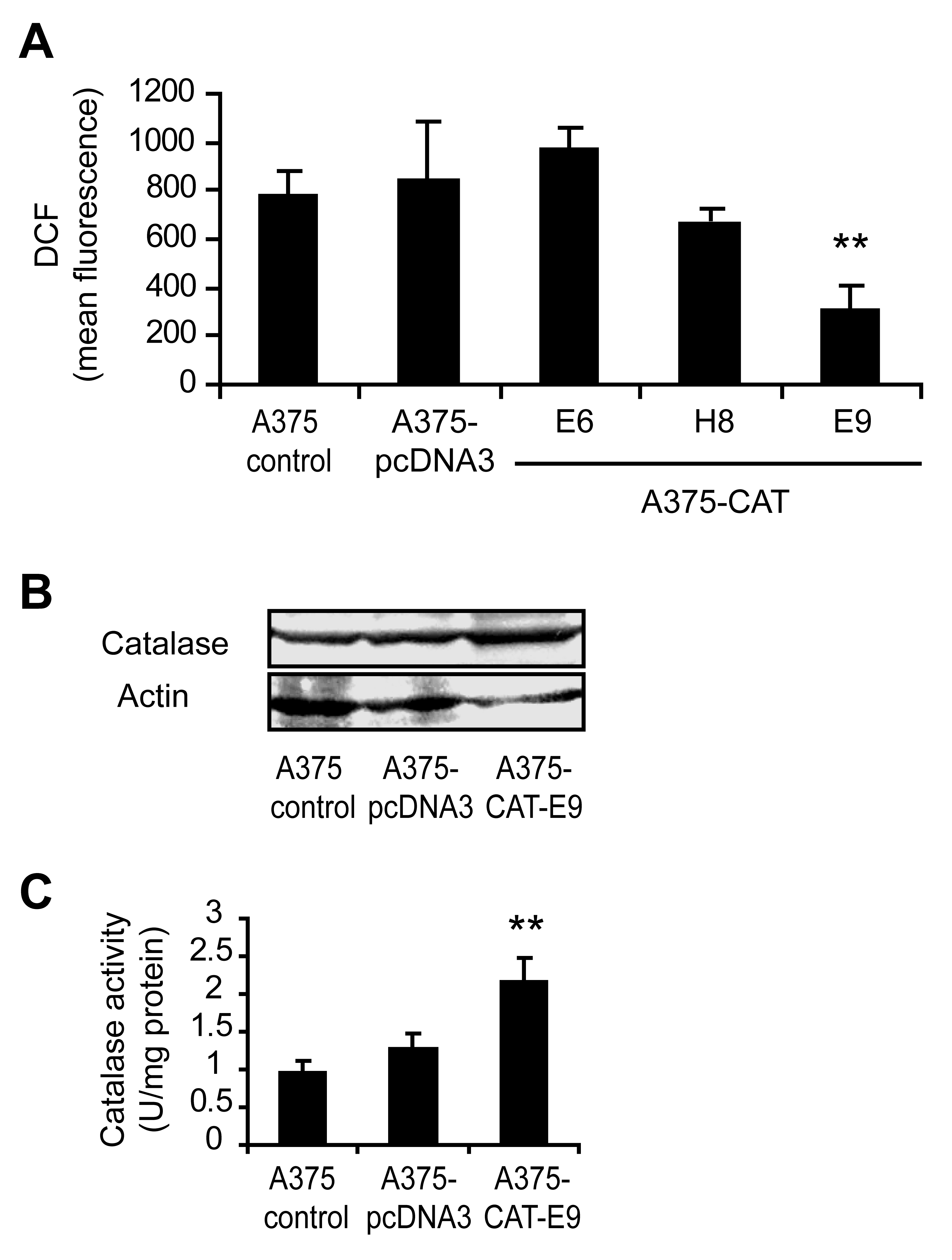

Supplement: Figure S1 — Characterization of the catalase-overexpression model. (A) Clone A375-CAT-E9 showed the lowest intracellular ROS levels of the stable geneticin-resistant clones generated. DCF mean fluorescence (arbitrary units) of A375 cells stably transfected with a construct containing the pcDNA3 expression vector and the cDNA coding for human catalase (A375-CAT). Control cells were either transfected with empty pcDNA3 vector (A375-pcDNA3) or left non-transfected (A375 control). (B) Increased levels of catalase in clone A375-CAT-E9 as compared with A375-pcDNA3 or A375 control, determined by western blot. (C) Higher catalase activity of A375-CAT-E9 cells than control ones (A375-pcDNA3 or A375 control). (A and C) Data are expressed as mean ± SD. **p<0.01 vs. A375 control. (TIF) [file pone.0044502.s001.tif]

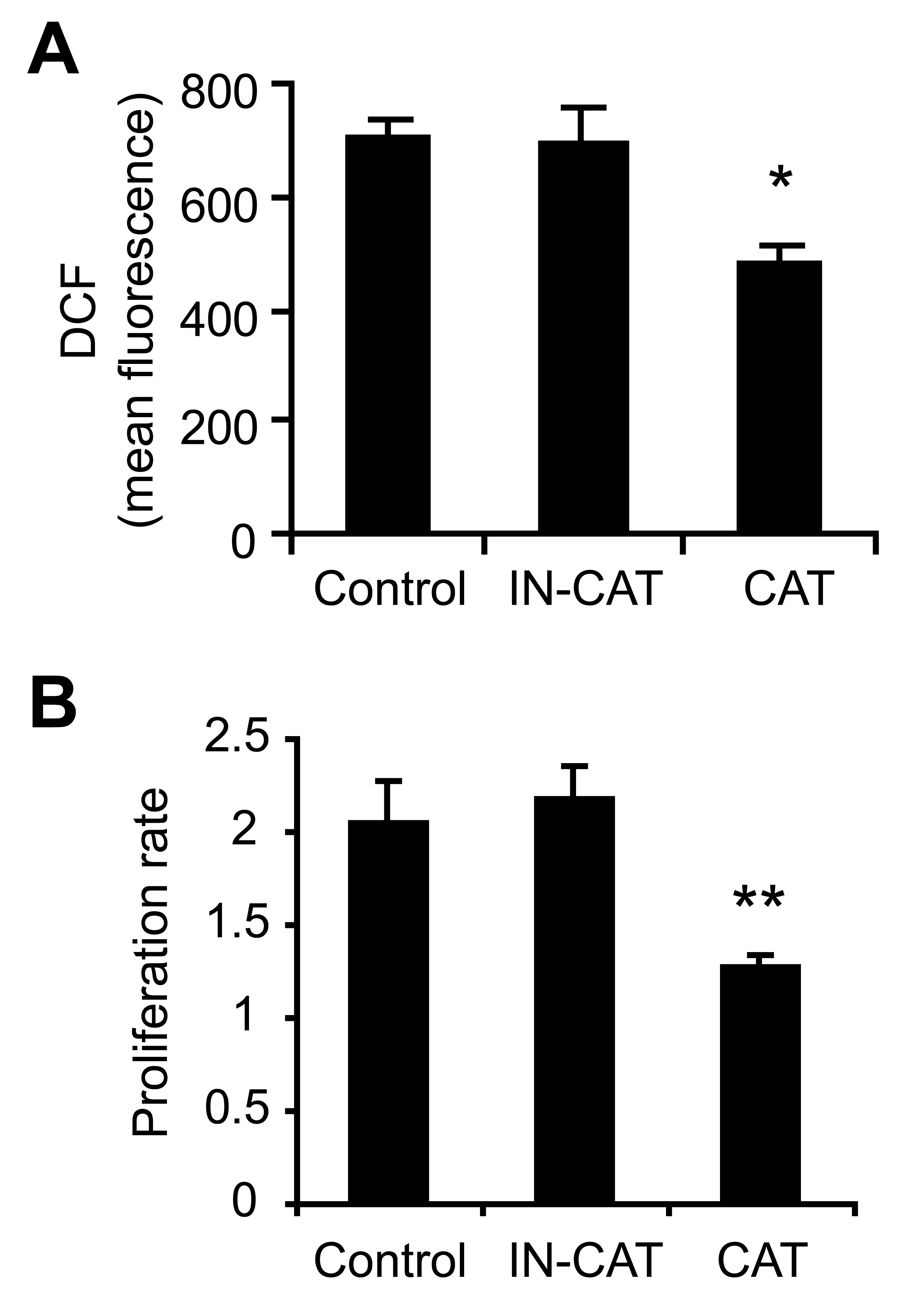

Supplement: Figure S2 — Cells treated with heat-inactivated catalase exhibited no significant differences with non treated cells. (A) The levels of ROS were measured by the DCFH-DA assay and (B) the proliferation rate by the MTT assay. A375 melanoma cells were treated with 1000 U/ml of catalase (CAT) or 1000 U/ml heat-inactivated catalase (IN-CAT) in PBS for 24 h or left untreated (control). Data are expressed as mean ± SD. *p<0.05 and **p<0.01 vs. A375 control. (TIF) [file pone.0044502.s002.tif]

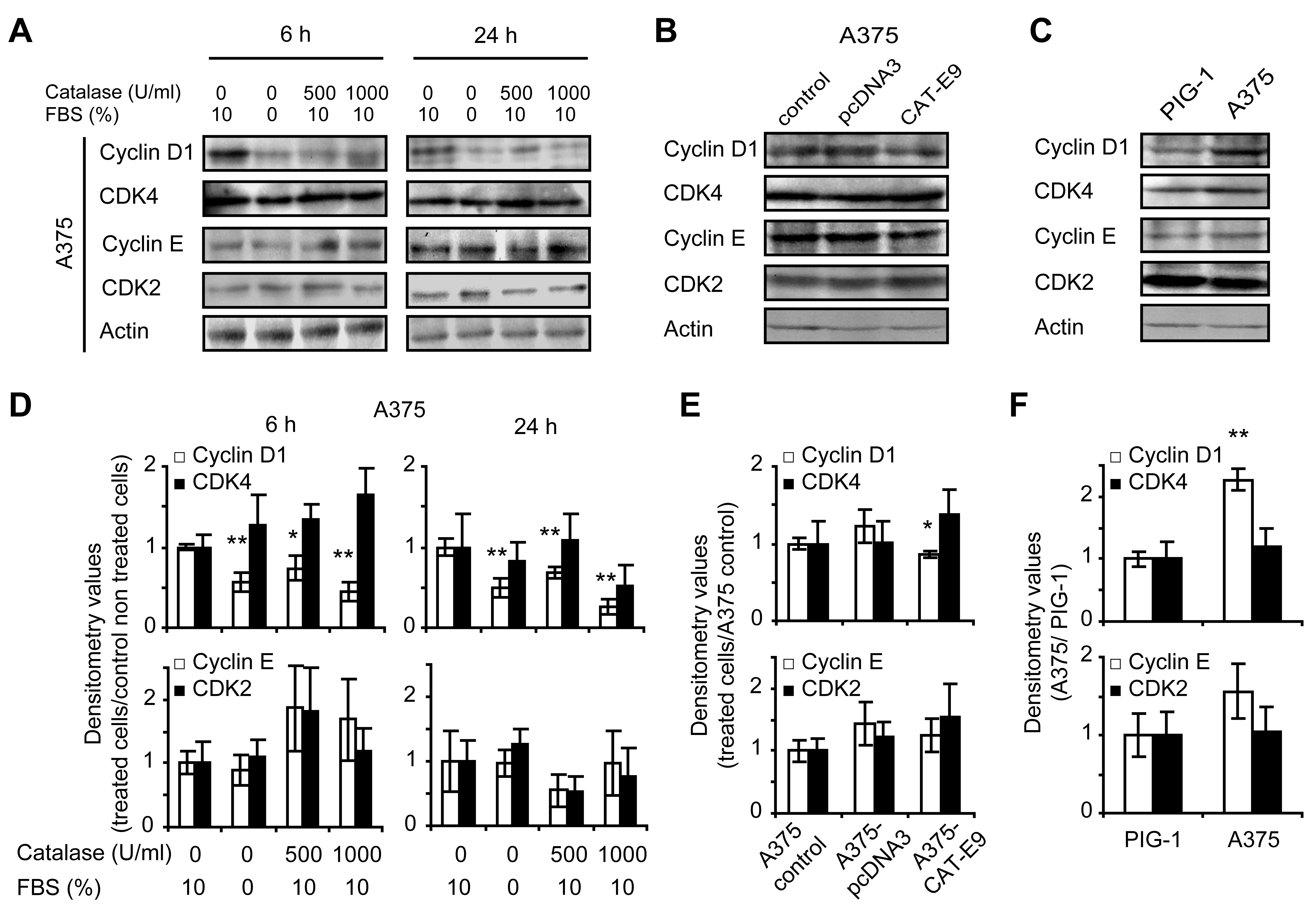

Supplement: Figure S3 — Cyclin D1 levels decreased in response to H2O2 scavenging and intrinsic low levels of H2O2. The expression of cyclins and CDKs of G1/S was analyzed by western blot. (A and D) Melanoma cells treated with catalase (CAT) for 6 and 24 h. FBS starved cells were used as control of G1 arrest. (B and E) Catalase overexpression model (A375-CAT-E9 cells) vs. controls (A375-pcDNA3 and A375 control cells). (C and F) Non-tumor (PIG-1) vs. tumor (A375) cells. (A–C) Representative western blot images. (D–F) Relative densitometric values of cyclins and CDKs. Actin densitometric values were used to standardize for protein loading. Data are expressed as mean ± SD. (D) *p<0.05 and **p<0.01 vs. control untreated. (E) *p<0.05 vs. A375 control (F) **p<0.01 vs. non-tumor cells. (TIF) [file pone.0044502.s003.tif]

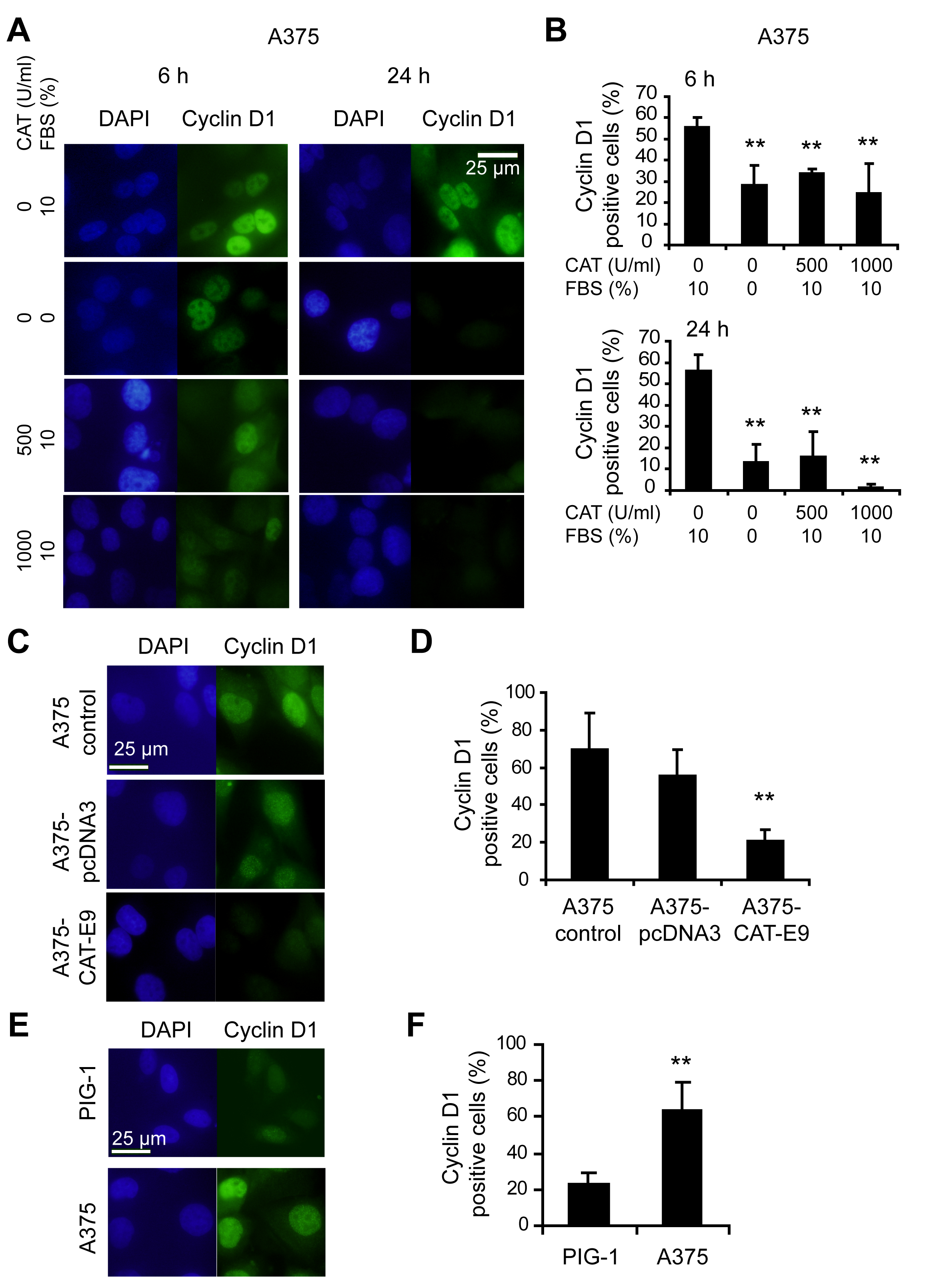

Supplement: Figure S4 — Immunocytofluorescence confirmed the decrease in cyclin D1 in response to catalase treatment in melanoma cells. Monoclonal anti-cyclin D1 (A-12, Santa Cruz Biotechnology) antibody, 1∶300 in PBS, and secondary FITC-conjugated anti-mouse IgG (Sigma) were used for immunocytofluorescence technique. (A–B) Melanoma cells treated with 500 and 1000 U/ml catalase (CAT) for periods of 6 or 24 h or left untreated. FBS starved cells were used as control of G1 arrest. (C–D) Catalase overexpression model (A375-CAT-E9 cells) vs. controls (A375-pcDNA3 and A375 control cells). (E–F) Non-tumor (PIG-1) vs. tumor (A375) cells. (A, C and E) Representative images of cyclin D1 immunocytofluorescence showing the subcellular localization of the protein. DAPI: staining of nuclear DNA; Cyclin D1: FITC staining of cyclin D1 protein. (B, D and F) Percentage of positive cells for cyclin D1 relative to the total number of counted cells. Data are expressed as mean ± SD. (B) **p<0.01 vs. untreated control. (D) **p<0.01 vs. A375 control. (F) **p<0.01 vs. non-tumor cells. (TIF) [file pone.0044502.s004.tif]

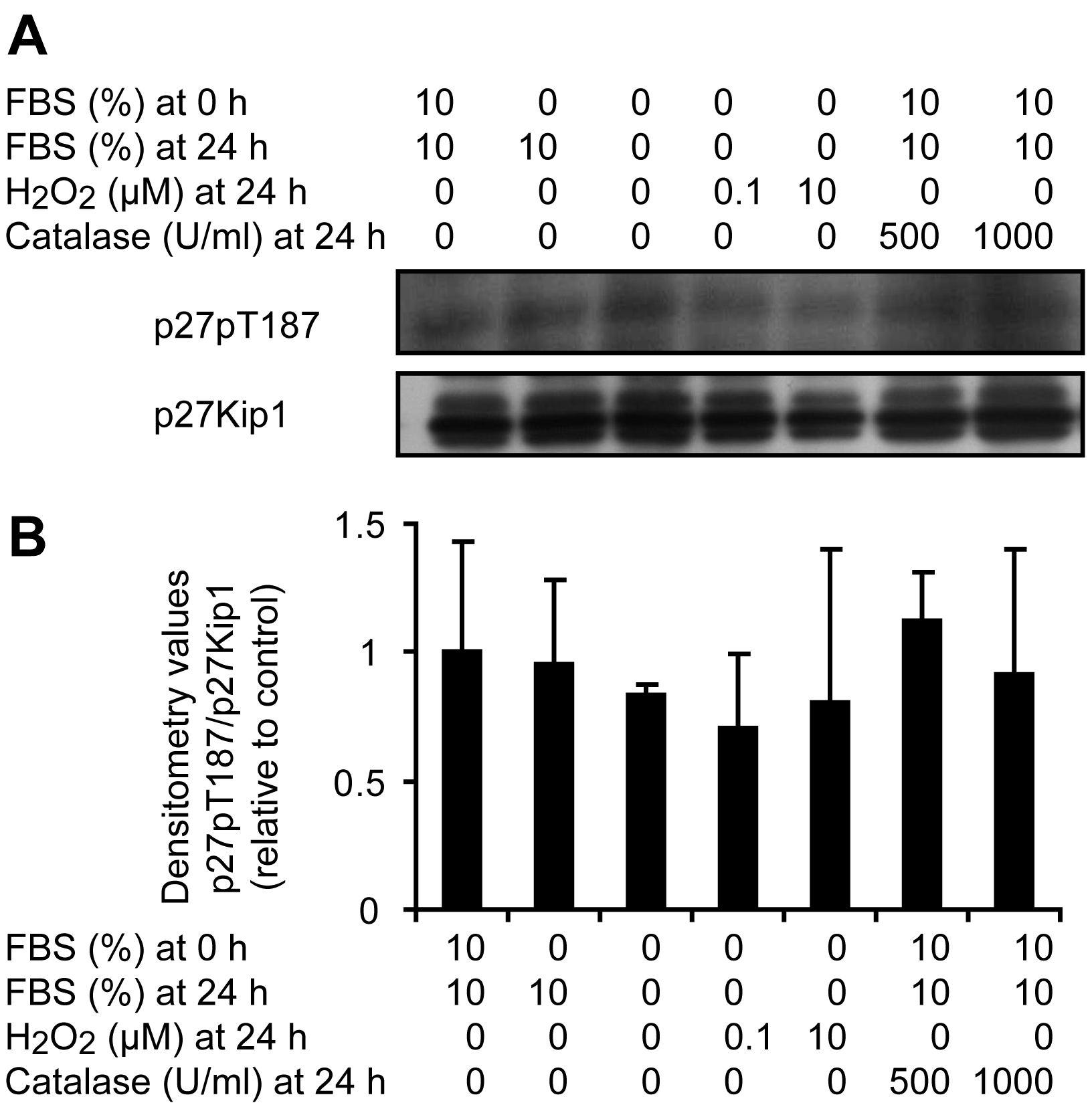

Supplement: Figure S5 — Phosphorylation of p27Kip1 on T187 is not modulated by H2O2 in melanoma cells. Melanoma (A375) cells grown in complete medium with 10% FBS were arrested by FBS starvation (0% FBS) for a period of 24 h or left untreated and then cells were incubated with different concentrations of H2O2 (0.1 or 10 µM) or to 10% FBS. Untreated cells were incubated with catalase 500 or 1000 U/ml. The expression of p27Kip1 and p27pT187 were analyzed by western blot. (A) Representative immunoblot images. (B) Relative densitometric values of p27pT187 referred to p27Kip1. Actin densitometric values were used to standardize for protein loading. Results are referred to control incubated with 10% FBS. (TIF) [file pone.0044502.s005.tif]

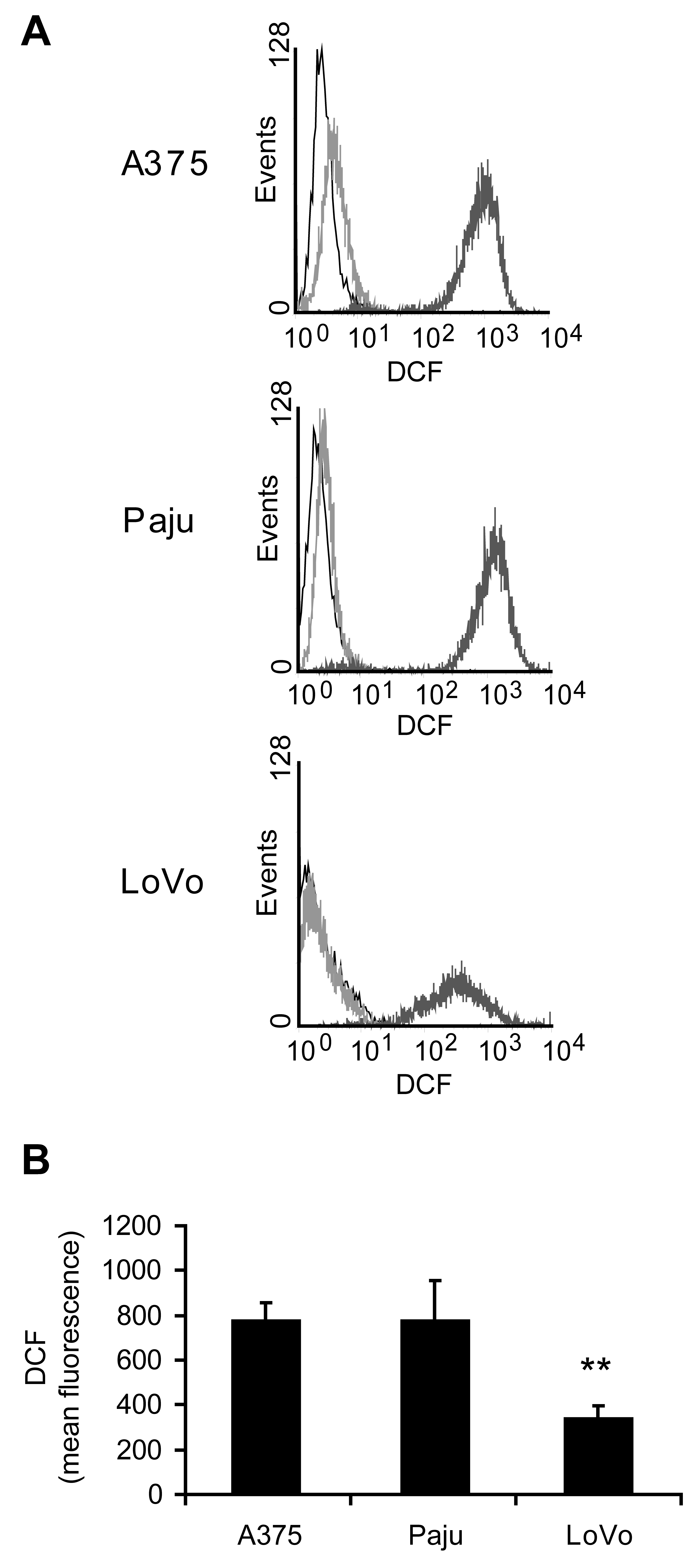

Supplement: Figure S6 — Intracellular ROS levels in tumor cells of different origin determined by DCFH-DA assay. Colorectal carcinoma cells (LoVo) exhibited lower intracellular ROS levels than neuroblastoma (Paju) and melanoma (A375) cells. (A) Representative histograms of DCF fluorescence: control cells not exposed to DCFH-DA (■), control cells treated with catalase just before DCFH-DA incubation () and cells incubated with DCFH-DA (). (B) DCF mean fluorescence (arbitrary units) of tumor cells. Data are expressed as mean ± SD. **p<0.01 vs. A375 cells. (TIF) [file pone.0044502.s006.tif]

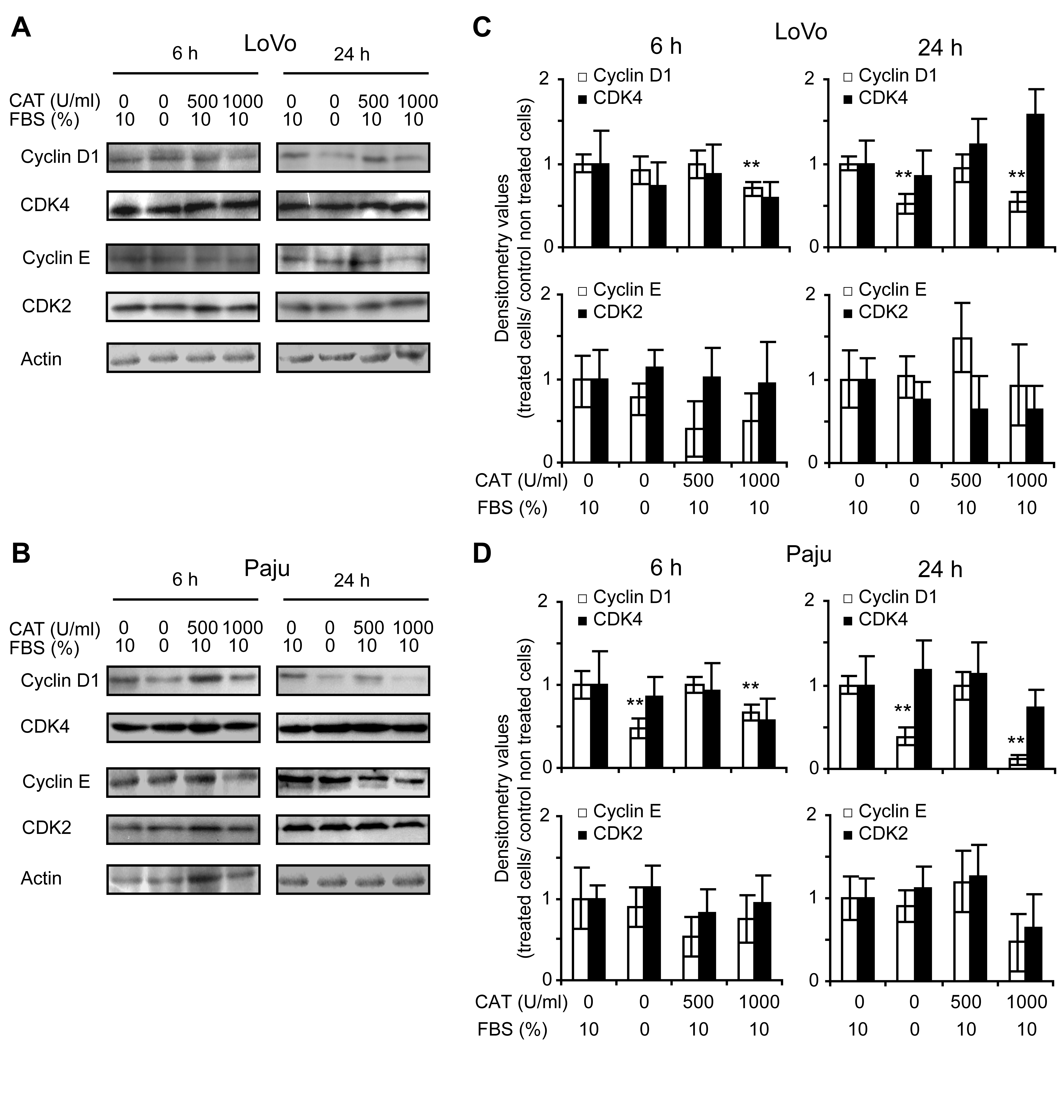

Supplement: Figure S7 — Decrease of cyclin D1 by catalase was also found in colon adenocarcinoma and neuroblastoma cells. The expression of cyclins and CDKs of G1/S was analyzed by western blot in (A and C) LoVo and (B and D) Paju cells treated with catalase (CAT) for 6 and 24 h. FBS starved cells were used as control of G1 arrest. (A and B) Representative western blot images. (C and D) Relative densitometric values of cyclins and CDKs. Actin densitometric values were used to standardize for protein loading. Data are expressed as mean ± SD. **p<0.01 vs. control untreated. (TIF) [file pone.0044502.s007.tif]

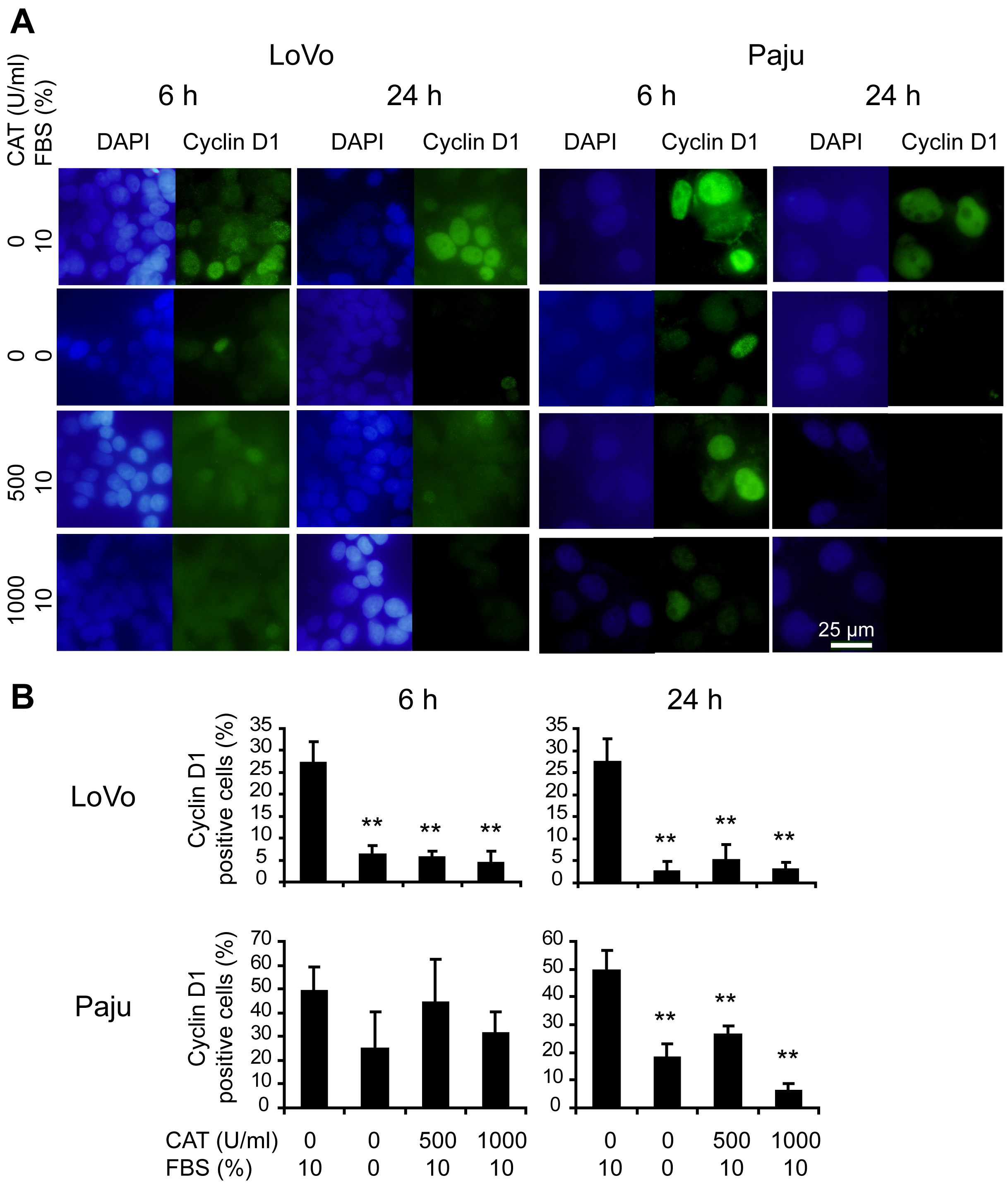

Supplement: Figure S8 — Low signal of cyclin D1 after catalase treatment in LoVo and Paju cells by immunocytofluorescence. See Methods S2 for immunocytofluorescence technique. (A) Representative images of cyclin D1 immunocytofluorescence showing the subcellular localization of the protein in tumor cells treated with 500 and 1000 U/ml catalase (CAT) for periods of 6 or 24 h compared to untreated controls. FBS starved cells were used as control of G1 arrest. DAPI: staining of nuclear DNA; Cyclin D1: FITC staining of cyclin D1 protein. (B) Percentage of positive cells for cyclin D1 relative to the total number of counted cells. Data are expressed as mean ± SD. **p<0.01 vs. untreated control. (TIF) [file pone.0044502.s008.tif]

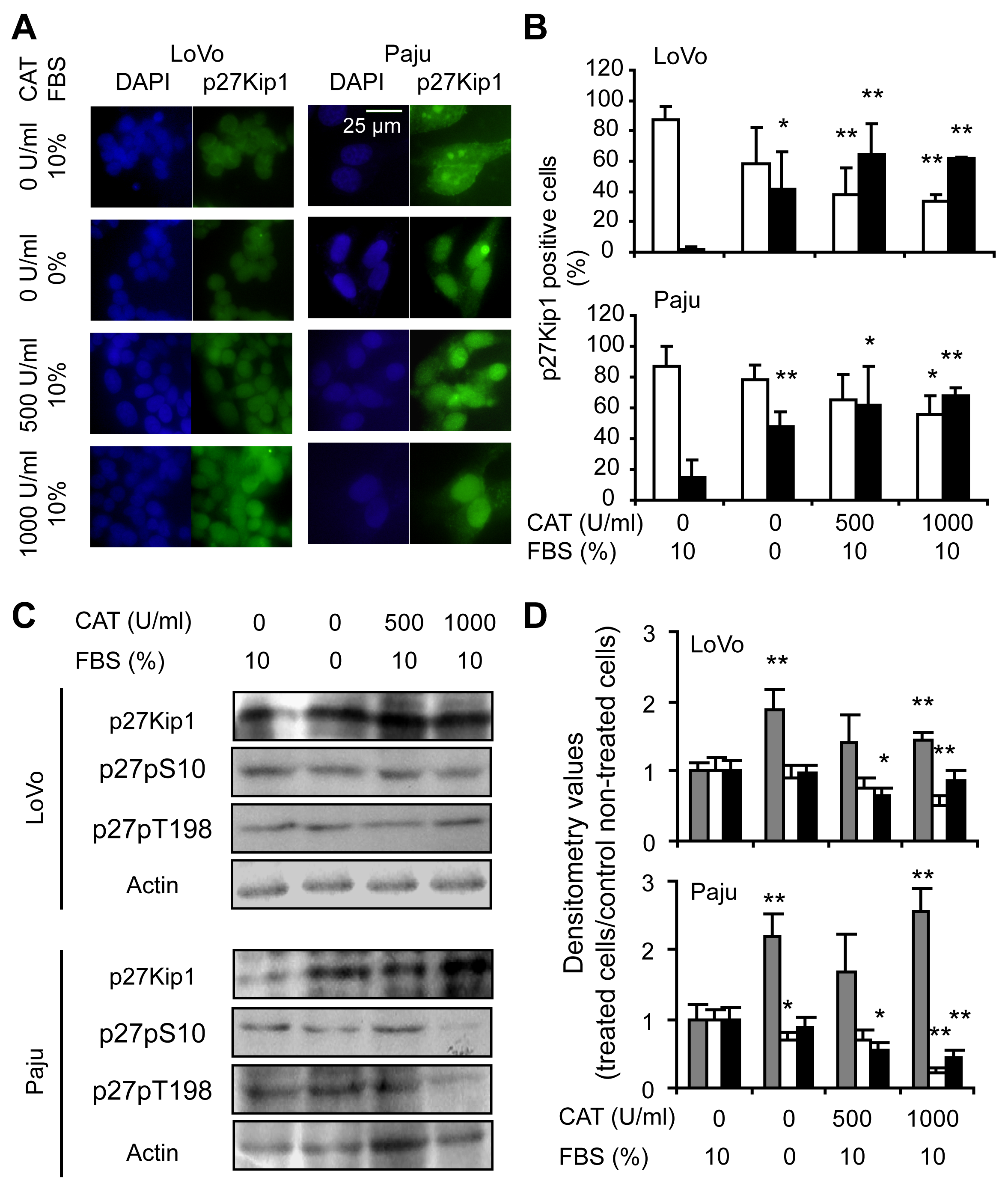

Supplement: Figure S9 — Relocalization of p27Kip1 in colon adenocarcinoma and neuroblastoma cells after 6 h of catalase treatment. (A and B) Nuclear localization of p27Kip1 induced by catalase (CAT) was detected by immunocytofluorescence. (A) Representative images of p27Kip1 immunocytofluorescence showing the subcellular localization of the protein. DAPI: staining of nuclear DNA; p27Kip1: FITC staining of p27Kip1 protein. (B) Percentage of positive cytoplasms (□) and positive nuclei (■) for p27Kip1 relative to the total number of counted cells. (C and D) Increase of p27Kip1 levels and decrease of p27Kip1 phosphorylated at S10 (p27pS10) and T198 (p27pT198) in response to H2O2 scavenging, analyzed by western blot. (C) Representative immunoblot images. (D) Relative densitometric values of () p27Kip1 levels, (□) p27pS10 and (■) p27pT198. Actin densitometric values were used to standardize for protein loading. Results are referred to control without treatment. (B and D) Data are expressed as mean ± SD. *p<0.05 and **p<0.01 vs. untreated control. (A–D) FBS starved cells were used as control of G1 arrest. (TIF) [file pone.0044502.s009.tif]
