# Supplementary material for: Phosphorylation and Subcellular Localization of p27Kip1 Regulated by Hydrogen Peroxide Modulation in Cancer Cells
Source: PLoS One. 2012 Sep 6;7(9):e44502. doi: 10.1371/journal.pone.0044502 (PMC3435274; doi:10.1371/journal.pone.0044502)
Supplement: Methods S1 — Catalase expression determination by western blot and measurement of catalase activity. (DOC) [file pone.0044502.s010.doc]

**Supplementary Methods S1**

In order to characterize the catalase-overexpression model, catalase expression and activity were determined. Cells were transfected with the CAT-pcDNA3 or with the empty vector or left untransfected.

**Catalase expression determination by Western Blot**

To obtain cell extracts, cells were incubated on ice for 30 min in RIPA lysis buffer (Sigma) containing the Halt protease and phosphatase inhibitor cocktail (Thermo Scientific). The protein yield was quantified by the DC Protein Assay Reagent (BioRad) based on the Lowry protocol. Samples were separated by SDS polyacrylamide (Promega) gel electrophoresis, transferred to nitrocellulose membranes (Hybond ECL Membrane, Amersham Biosciences, GE Healthcare) and immunoblotted by appropriate antibodies.

The antibody against catalase was purchased from Novocastra. The primary antibody was detected using horseradish peroxidase-linked donkey anti-rabbit IgG (Amersham, GE Healthcare) and visualized by the ECL detection system (Amersham Biosciences, GE Healthcare). Actin detection was performed to show samples loading. Three independent experiments were performed with duplicates per experimental condition.

**Measurement of Catalase Activity**

Catalase activity was determined using a spectrophotometric assay as described by Aebi et al. [1]. Briefly, cells grown in 100-mm dishes at 70% confluence were scraped, homogenized in 200 μl phosphate buffer 50 mM, pH 7.2 and sonicated. The homogenates were centrifuged at 14,000 rpm. Protein concentrations were measured using the DC Protein Assay Reagent (BioRad) based on the Lowry protocol. One milliliter of phosphate buffer 50 mM, pH7 containing H_2_O_2_ 30 mM was mixed with cell extracts to start the reaction. The decomposition of H_2_O_2_ was followed directly by the decrease in absorbance at 240 nm, which was monitored spectrophotometrically. A unit of catalase is defined as the disappearance of 1 μmol H_2_O_2_/min at 25 ºC (E= 39.4 M^-1^ cm^-1^). Three independent experiments were performed with triplicates per condition.

**References**

1. Aebi H (1984) Catalase in vitro. Methods Enzymol. 105: 121-126.
