# Supplementary material for: Phosphorylation and Subcellular Localization of p27Kip1 Regulated by Hydrogen Peroxide Modulation in Cancer Cells
Source: PLoS One. 2012 Sep 6;7(9):e44502. doi: 10.1371/journal.pone.0044502 (PMC3435274; doi:10.1371/journal.pone.0044502)
Supplement: Methods S2 — Detection of cyclin D1 by immunocytofluorescence. (DOC) [file pone.0044502.s011.doc]

**Supplementary Methods S2**

**Detection of cyclin D1 by immunocytofluorescence**

Subconfluent cell cultures grown in 60 mm dishes were fixed in 4% (w/v) paraformaldehyde in PBS for 15 min. Cells were then washed with PBS, permeabilized with 0.5% (v/v) Triton X-100 in PBS for 15 min, washed and blocked with 5% (v/v) FBS in PBS for 30 min. Cells were incubated overnight at 4ºC with the monoclonal anti-cyclin D1 (A-12, Santa Cruz Biotechnology) antibody, 1:300 in PBS, washed and incubated with secondary FITC-conjugated anti-mouse IgG (Sigma) for 1 h in the dark at room temperature. Finally, the samples were washed, counterstained and mounted with 1 µg/ml 4’,6-diamidine-2’-phenylindole (DAPI, Sigma) in an antifade solution in the dark. Cells were examined in an Olympus BX51 epifluorescence microscope utilizing immersion oil with a 100X (UPlanApo 100 X/1.35 oil) objective lens. For each treatment condition, FITC and DAPI images were serially captured by a CCD camera (Olympus DP70) and more than 50 fields containing approximately 20 cells each were stored. A code number was given to each image. Random sampling methods were used to select the images and all the cells in each selected image were screened. An average of 250 cells was evaluated per experimental condition. Total and positive cells, for cyclin D1 were counted by eye by two scorers and results were crosschecked. Three independent experiments were performed with triplicates per condition.
