# Supplementary material for: Phosphorylation and Subcellular Localization of p27Kip1 Regulated by Hydrogen Peroxide Modulation in Cancer Cells
Source: PLoS One. 2012 Sep 6;7(9):e44502. doi: 10.1371/journal.pone.0044502 (PMC3435274; doi:10.1371/journal.pone.0044502)
Supplement: Methods S3 — Determination of G1/S regulatory proteins and p27Kip1 phosphorylated at T187 by western blot. (DOC) [file pone.0044502.s012.doc]

**Supplementary Methods S3**

**Determination of G1/S regulatory proteins and p27Kip1 phosphorylated at T187 by western blot**

Cells were treated with 500 or 1000 U/ml catalase or left untreated for 6 or 24 h for determination of G1/S regulatory proteins. Cells were treated with H_2_O_2_ (0.1 or 10 µM) or with catalase (500 or 1000 U/ml) or left untreated for 24 h for determination of p27pT187. FBS starved cells were used as control of G1 arrest. To obtain cell extracts, cells were incubated on ice for 30 min in RIPA lysis buffer (Sigma) containing the Halt protease and phosphatase inhibitor cocktail (Thermo Scientific). The protein yield was quantified by the DC Protein Assay Reagent (BioRad) based on the Lowry protocol. Samples were separated by SDS polyacrylamide (Promega) gel electrophoresis, transferred to nitrocellulose membranes (Hybond ECL Membrane, Amersham Biosciences, GE Healthcare) and immunoblotted by appropriate antibodies.

The antibodies against cyclin D1 (C-20), CDK4 (C-22), cyclin E (M-20), CDK2 (M-2), p27pT187 (Thr 187) and actin (I-19) were purchased from Santa Cruz Biotechnology. The primary antibodies were detected using horseradish peroxidase-linked donkey anti-rabbit IgG (Amersham, GE Healthcare) or anti-goat IgG (Santa Cruz Biotechnology) and visualized by the ECL detection system (Amersham Biosciences, GE Healthcare). Three independent experiments were performed with duplicates per experimental condition.
